# *De novo* assembly of a transcriptome for the cricket *Gryllus bimaculatus* prothoracic ganglion: An invertebrate model for investigating adult central nervous system compensatory plasticity

**DOI:** 10.1371/journal.pone.0199070

**Published:** 2018-07-11

**Authors:** Harrison P. Fisher, Micah G. Pascual, Sylvia I. Jimenez, David A. Michaelson, Colby T. Joncas, Eleanor D. Quenzer, Andrew E. Christie, Hadley W. Horch

**Affiliations:** 1 Department of Biology, Bowdoin College, Brunswick, Maine, United States of America; 2 Békésy Laboratory of Neurobiology, Pacific Biosciences Research Center, School of Ocean and Earth Science and Technology, University of Hawaii at Manoa, Honolulu, Hawaii, United States of America; Federal University of Rio de Janeiro, BRAZIL

## Abstract

The auditory system of the cricket, *Gryllus bimaculatus*, demonstrates an unusual amount of anatomical plasticity in response to injury, even in adults. Unilateral removal of the ear causes deafferented auditory neurons in the prothoracic ganglion to sprout dendrites across the midline, a boundary they typically respect, and become synaptically connected to the auditory afferents of the contralateral ear. The molecular basis of this sprouting and novel synaptogenesis in the adult is not understood. We hypothesize that well-conserved developmental guidance cues may recapitulate their guidance functions in the adult in order to facilitate this compensatory growth. As a first step in testing this hypothesis, we have generated a *de novo* assembly of a prothoracic ganglion transcriptome derived from control and deafferented adult individuals. We have mined this transcriptome for orthologues of guidance molecules from four well-conserved signaling families: Slit, Netrin, Ephrin, and Semaphorin. Here we report that transcripts encoding putative orthologues of most of the candidate developmental ligands and receptors from these signaling families were present in the assembly, indicating expression in the adult *G*. *bimaculatus* prothoracic ganglion.

## Introduction

Within the spectrum of adult neuronal plasticity, large-scale anatomical plasticity is often less common than other types of plasticity, such as changes in synaptic strengths, reorganization of receptive fields, and alterations of dendritic spine morphology [1,2]. We are particularly interested in the subset of plasticity related to anatomical reorganization of neuronal structures in the adult central nervous system (CNS) in response to sensory loss. In contrast to many mammalian models that demonstrate little neuronal plasticity in adulthood [3,4], invertebrates are often capable of large-scale anatomical plasticity at all stages of their life history [2,5,6]. The present study focuses on the Mediterranean field cricket, *Gryllus bimaculatus*, as a model of anatomical plasticity during adulthood. More specifically, the cricket can be utilized to study the molecular control of the compensatory dendritic growth of deafferented auditory neurons in the CNS.

**Fig 1:**
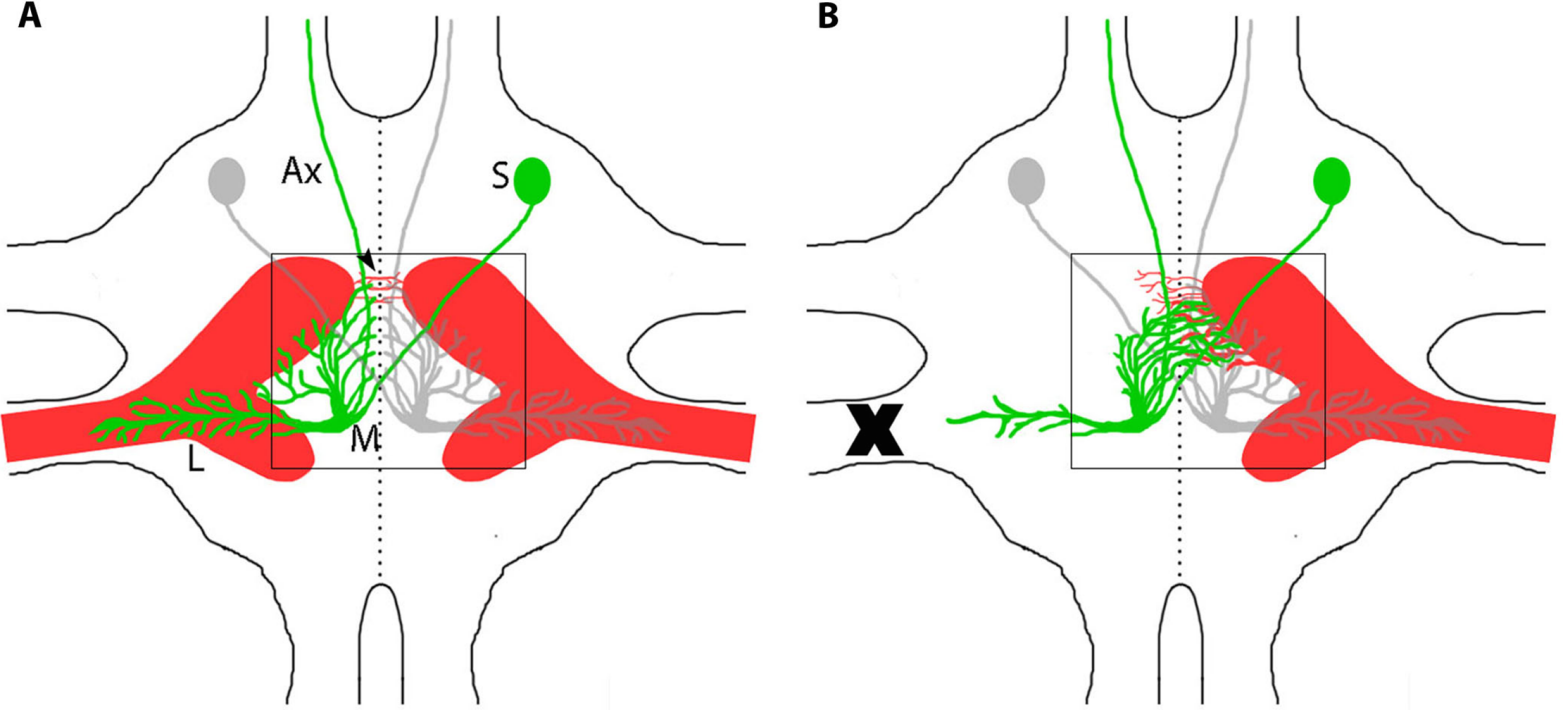
The central auditory system of adult *G*. *bimaculatus* shows large-scale compensatory anatomical plasticity after injury. A) Auditory afferents (red “claw”) terminate in the prothoracic ganglia, and synapse with ascending neurons (AN-2 shown in green and gray). AN-2 neurons have lateral dendrites (L) and medial dendrites (M) that grow up to but not across the midline (dashed line). AN-2 soma (S) are on the opposite side of the midline from the dendrites, and its axons (Ax) projects up to the brain. B) After unilateral loss of an ear, afferents degrade (X), leaving the ipsilateral ANs deafferented. AN-2 dendrites sprout across the midline and become synaptically connected to the contralateral afferents, compensating for the loss of the ear. Fig adapted from [13].

In *G*. *bimaculatus*, the prothoracic ganglion exhibits compensatory plasticity in response to CNS injury. Bilateral ascending neurons, AN-1 and AN-2, are pairs of auditory neurons located on either side of the midline in the prothoracic ganglion that process auditory input and send it to the brain (Fig 1A). AN-2 displays sensitivity to high frequency auditory stimuli above 12kHz, such as bat calls [7], and bursting of AN-2 elicits escape behavior during flight. The dendritic arbor of each AN-2 is localized on either side of the midline and receives input from the ipsilateral nerve 5 (Fig 1A), which delivers sensory information from the ipsilateral auditory organ, located on the foreleg. In several mammalian auditory systems, plastic changes occur in response to manipulation during development, but not during adulthood [8–10]. In contrast, the prothoracic auditory system in the cricket maintains plasticity into adulthood [11]. Removing auditory input to AN-2 (deafferentation) induces the AN-2 dendrites to grow across the midline, a boundary rarely crossed by innervated dendrites, and make functional synaptic connections with the contralateral auditory afferents (Fig 1B). This compensatory anatomical response is evident both during development [12] and in adulthood [11,13]. The evidence of anatomical neuronal plasticity in adults provides a unique and interesting point of inquiry into if and how mechanisms of plasticity shift between development and adults. Furthermore, the molecular control of this compensatory growth remains largely unknown because of the relative scarcity of genomic/transcriptomic information available for *G*. *bimaculatus*.

Although *G*. *bimaculatus* is less well-studied than other model systems, it nonetheless has a rich neuroethological experimental history c.f. [14], and was first used as a model for exploring the compensatory plasticity of the central nervous system over 30 years ago [12]. Crickets, along with other orthopteroid insect orders including grasshoppers, roaches, termites, earwigs, and praying mantises, belong to the Polyneoptera supercohort of the Insecta [15]. Recent work by Misof and colleagues (2014) estimates the origin of the Polyneoptera to be around 302 million years ago (Ma). More specifically, the Orthoptera diverged around 200Ma; the Coleoptera diverged at approximately 240Ma, and *Tribolium* around 120Ma. By comparison, Diptera were purported to originate approximately 150Ma with *Drosophila* diverging approximately 85Ma [15]. Thus, *G*. *bimaculatus* serves as an evolutionary intermediate between more basal models, such as the nematode *Caenorhabditis elegans*, and the more recently diverged insects *Tribolium* and *Drosophila*. Advantages to the cricket as a model organism include its ease of culturing in lab, as well as the development of several sophisticated tools for assessing and altering gene expression or protein levels, [16–21]. In the present study, we describe the generation of a *de novo* transcriptome for the *G*. *bimaculatus* adult prothoracic ganglion, a resource that we have used to assess the presence of developmental guidance cues in adult tissue. Though two previous transcriptomes have been assembled from *G*. *bimaculatus* tissue [18,19], this is the first derived solely from adult neuronal tissue, and specifically from the adult prothoracic ganglion. Converging lines of evidence from other animal models suggest that anatomical neuronal plasticity during adulthood may involve the recapitulation of the guidance cues that direct neurite growth during development [22,23]. Although most of these guidance cues were originally explored for their roles in axonal guidance, many have been implicated in dendritic guidance as well [24–26]. During development, secreted guidance cues create a molecular environment of appropriately placed attractive and repulsive cues, often expressed in gradients, that function to guide developing neurites and establish boundaries such as the midline [26–29]. These secreted cues exert different effects on developing neurons depending on the type of receptor activated and/or the concentration of the intracellular signaling molecules in question [28,30–33]. Presumably, once neurites have reached their target locations, many of the guidance cues are no longer needed, or they establish an environment that simply maintains mature axonal and dendritic structures[28]. Of all the major guidance cue families, *i*.*e*., Slit, Semaphorin, Netrin, and Ephrin, none have been observed to maintain the same developmental expression distributions into adulthood [28]. However, in response to profound challenges to the adult nervous system, such as injury or sensory loss, the molecular environment of guidance cues might be altered to promote or enable anatomical plasticity.

As an initial step in assessing potential alterations in guidance cue functions in response to injury, a *de novo* transcriptome was constructed from Illumina sequencing reads of RNA extracted from male cricket prothoracic ganglia. In order to assess the recapitulation hypothesis, that expression patterns of developmental guidance cues are altered as a mechanism for restoring or enhancing plasticity in the adult CNS, adult crickets were used to generate this transcriptome. All crickets had a portion of their legs amputated, either distally at the tarsus (“foot chop” control), or more proximal at the distal end of the femur, so the auditory organ on the tibia was removed (“deafferented” experimental), before the neuronal tissue was removed for the RNA isolation. This transcriptome, which consists of 511,724 transcripts, has been deposited publically (**BioProject No.** **PRJNA376023**) for use as a resource for future studies of the prothoracic ganglion, and neuronal signaling in *G*. *bimaculatus* in a general sense. Because minimal genomic information is known about *G*. *bimaculatus*, the present study seeks to determine the representation of known guidance cues and related proteins in the adult cricket by constructing a predicted proteome. Protein sequences corresponding to members of the main families of guidance cues in *Drosophila* were used to search for matching sequences in our *de novo* transcriptome. We hypothesize that the male adult cricket prothoracic ganglion contains members of most known developmental guidance cue families, and thus will be present in our transcriptome. Expression of these proteins in adult control or deafferented prothoracic ganglia could indicate that this ganglion is an area of neuronal tissue that maintains or is capable of reinstating neuronal plasticity.

## Results and discussion

### *De novo* assembly of a prothoracic ganglion-specific transcriptome

In the present study, next generation sequencing on individual prothoracic ganglia of the Mediterranean field cricket was performed using the Illumina HiSeq platform. Twenty-one samples, each consisting of the prothoracic ganglion from a single adult male cricket in a control, deafferented, or backfill condition (see methods), were used as the source of RNA for transcriptome development (Table 1). RNA-Seq yielded approximately 56 million paired-end reads per library (Table 1), which were collectively assembled using Trinity; a total of 751,876,742 trimmed and quality filtered paired-end reads (100 bp in length) were input into Trinity for *de novo* assembly (Table 2). The transcriptome assembled by Trinity consisted of 511,724 transcripts and 374,383 “Trinity predicted genes” (Table 2). Of the “Trinity predicted genes”, 327,675 were singletons (87.5%), and the remaining genes (46,508 in total) possessed from two to 41 “Trinity predicted isoforms”. The average transcript length was 897 bp (Table 2); the N50 was 1,981 bp (Table 2), with the shortest and longest assembled sequences being 200 and 44,326 bp, respectively (Table 2). Mapping for the Illumina-generated reads against the complete 511,724-transcript assembly using Bowtie2 yielded an overall alignment rate of 99.53% (Table 3). There were an unusually high percentage of reads that mapped more than one time (96.89%). This high multi-mapping percentage, along with the high number of contigs, is likely explained by oversampling [34]and the fact that this was a *de novo* assembly, not one that used a genome for scaffolding [35]. Much of the data discussed and mined from the transcriptome is included in the first supplemental figure. The complete, quality filtered dataset (509,779 of the original 511,724 contigs), is available at the NCBI under **BioProject No.** **PRJNA376023**.

**Table 1:** Summary of *G*. *bimaculatus* prothoracic ganglia samples and their Illumina sequencing.

Sample	Condition	Total RNA Concentration (ng/μl)	Raw Reads* (PF PE)
**1**	24hr control	17.3	54,623,249
**2**	24hr control	17.1	60,237,531
**3**	24hr control	16.8	57,092,895
**4**	24hr deafferent	25	63,734,867
**5**	24hr deafferent	23.6	55,569,346
**6**	24hr deafferent	24.3	47,602,610
**7**	3-day control	22.1	42,256,990
**8**	3-day control	34.4	56,725,824
**9**	3-day control	14.9	56,514,006
**10**	3 day deafferent	26.2	58,703,677
**11**	3 day deafferent	25.2	60,193,146
**12**	3 day deafferent	39.3	63,232,759
**13**	7-day control	14.8	40,423,286
**14**	7-day control	34	31,114,705
**15**	7-day control	27.5	64,312,361
**16**	7 day deafferent	28.7	57,027,250
**17**	7 day deafferent	18.9	56,265,370
**18**	7 day deafferent	38.9	60,236,625
**19**	24 hour backfill	60	58,069,130
**20**	24 hour backfill	35.5	66,478,056
**21**	24 hour backfill	43.2	69,922,928

**Table 2:** *G*. *bimaculatus* prothoracic ganglion transcriptome assembly statistics.

**Total number of bases assembled**	**237,416,984**
**Total number of reads assembled**	**751,876,742**
**Total number of assembled contigs**	**511,724**
**Total number of Trinity “genes”**	**374,383**
**Minimum contig length (bp)**	**200**
**Maximum contig length (bp)**	**44,326**
**Average contig length (bp)**	**897.08**
**Median contig length (bp)**	**381**
**N50 (bp)**	**1981**
**Total GC count (bp)**	**95,774,011**
**GC content for the complete assembly (%)**	**40.34**

Transcriptome assembly statistics were generated using Trinity software. Reads used for the de novo assembly were trimmed for Illumina adapters and quality filtered. Read length = 100bp.

**Table 3:** Summary of the results of mapping RNA-Seq reads to the complete *G*. *bimaculatus* prothoracic ganglion assembly.

**Total reads used for mapping**	**751,876,742**
Total mapped reads*	**748,342,921**
**Overall alignment (%)**	**99.53**
**Reads mapped 1 time (#)**	**5,480,513**
**Reads mapped 1 time (%)**	**0.73**
**Reads mapped >1 time (#)**	**728,458,211**
**Reads mapped >1 time (%)**	**96.89**

*100% of the mapped reads aligned as clusters (read pairs)

This is the first prothoracic ganglion-specific transcriptome produced for a cricket; previous work in *G*. *bimaculatus* generated a transcriptome from developmental tissue [19], but was not specific to the nervous system. Similarly, a *de novo* transcriptome for *Gryllus firmus* was created from flight muscles and the fat body, but not neuronal tissue [36]. The public availability of the present dataset, and the associated raw data, provides a resource for assessing the molecular control of a system that displays a unique form of adult neural plasticity in response to auditory injury.

### *In silico* identification of developmental guidance cues in the adult prothoracic ganglion

The adult *G*. *bimaculatus* prothoracic ganglion-specific transcriptome just described was mined for transcripts encoding 25 different proteins (ligands, receptors, and intracellular signaling molecules) implicated in axonal and dendritic guidance; sequences for 21 of these were discovered in the assembly (summarized in Table 4). Top hits were translated into predicted proteins (translated sequences for all top hits are reported in S1 Fig) and evaluated for similarity to *Drosophila* proteins, since *Drosophila* represent the best described insect. Predicted proteins were also evaluated for the presence of various functional protein domains. Of the proteins identified, very few have been previously described in *G*. *bimaculatus*. Although transcripts covering full length sequences were found for only 13 of the queried proteins, translation of sequences and conserved domain analysis indicated that most sequences obtained were likely close to full-length. The presence of several proteins from these families in the adult cricket prothoracic ganglion raises the intriguing possibility that guidance cues may be involved in the compensatory response to injury, potentially through maintained plasticity or recapitulation of developmental programs.

**Table 4:** Developmental guidance molecules mined from adult prothoracic transcriptome.

Guidance Factor	Trinity ID	• Predicted Protein Portion • Length	• Most Similar *Drosophila* Counterpart • Accession No	% identity with *Drosophila* Counterpart
Slit	TRINITY_DN152282_c0_g1_i2	• Full-length • 1,459 AA	• Slit-F • AGB93549	63.5%
Robo1	TRINITY_DN172482_c0_g4_i2	• Full-length • 1,602 AA	• Robo1-C • AAM71113	40.2%
Robo2/3	TRINITY_DN174950_c3_g2_i1	• Internal • 1,340 AA	• Robo3-B • AGB92403	40.3%
Commisureless	TRINITY_DN156272_c0_g2_i3	• Internal • 145 AA	• Comm3-E • AHN58097	25.0%
Netrin-A	TRINITY_DN160784_c2_g1_i3	• C-Terminal • 340 AA	• Netrin-A-C • AHN59713	48.3%
Frazzled	TRINITY_DN172587_c0_g1_i7	• Full-length • 1,331 AA	• Frazzled-A • AAF58493	40.7%
Unc-5	TRINITY_DN149614_c0_g1_i1	• Internal • 254 AA	• Unc-5-B • AHN56248	37.1%
Dscam	TRINITY_DN173597_c2_g22_i1	• C-Terminal • 1,251 AA	• Dscam1-Y • ABI31042	66.8%
Ephrin	TRINITY_DN159904_c0_g1_i6	• C-Terminal • 307 AA	• Ephrin, isoform C • AAN06500	36.1%
Eph	TRINITY_DN159184_c0_g1_i3	• Full-Length • 996 AA	• Eph isoform E • AAN06503	59.5%
Sema1a	TRINITY_DN169697_c2_g1_i3	• Internal • 782 AA	• Sema-1a-G • AGB92801	59.5%
Sema2a	TRINITY_DN173773_c2_g2_i6	• Full-Length • 726 AA	• Sema-2a-E • AAS64837	69.4%
Sema5	TRINITY_DN165105_c1_g2_i8	• Full-Length • 1,057 AA	• Sema-5c-B • ACZ94701	44.4%
Plexin A	TRINITY_DN159247_c0_g1_i2	• Full Length • 1,909 AA	• Plexin A-A • AAF59394	69.9%
Plexin B	TRINITY_DN161683_c0_g1_i2	• Full Length • 1,850 AA	• Plexin B-B • AFH06760	60.1%
Off-track	TRINITY_DN160729_c0_g1_i15	• Full -Length • 538 AA	• Off-track-A • AAF58596	27.2%
Guanylyl Cyclase at 76C	TRINITY_DN166112_c0_g1_i2	• Internal • 1,135 AA	• Guanylyl Cyclase at 76C-D • AGB94756	58.1%
Mical	TRINITY_DN175376_c0_g1_i11	• Full-Length • 2,614 AA	• Mical-P ALI30552	19.2%
PKA-R2	TRINITY_DN165220_c0_g2_i1	• Full-Length • 382 AA	• PKA-R2-B • AAM71046	64.6%
Rac1	TRINITY_DN170604_c0_g3_i2	• Full-Length • 192 AA	• Rac1-B • AGB93942.1	94.8%
Rho1	TRINITY_DN165708_c0_g2_i4	• Full-Length • 192 AA	• Rho1-A • AAM70944	94.3%

*Drosophila melanogaster* query proteins used for tblastn searches were: Slit, (**AAF58098**) Robo 1, Isoform A (**AAF46887**), Robo 2 Isoform A (**AAF51375**), Robo 3, Isoform A (**AAF51387**), Commisureless, Isoform A (AAF49584.1), Netrin-A, Isoform A (**AAF48380**), Frazzled, Isoform A (**AAF58493**), Unc-5, Isoform A (**AAF58143**), Dscam, Isoform AE (**ABI31080**), Ephrin, Isoform A (**AAF59335**), Eph receptor tyrosine kinase, isoform A (**AAG22122**), Sema1a, Isoform A (**AAF52696**), Sema2a, Isoform A (**AAF57989**), Sema5c, Isoform A (**AAF49966**), Plexin A, Isoform A (**AAF59394**), Plexin B, Isoform A (**AAF59374**), Off-track, Isoform A (**AAF58596**), Mical, Isoform A (**AAF54478**), protein kinase, cAMP-dependent, regulatory subunit type 2, isoform A (**AAF58862**), Rac1 Isoform A (**AAF47469**), Rho 1 (**AAA67042)**.

### Slit and Robo signaling

**Fig 2:**
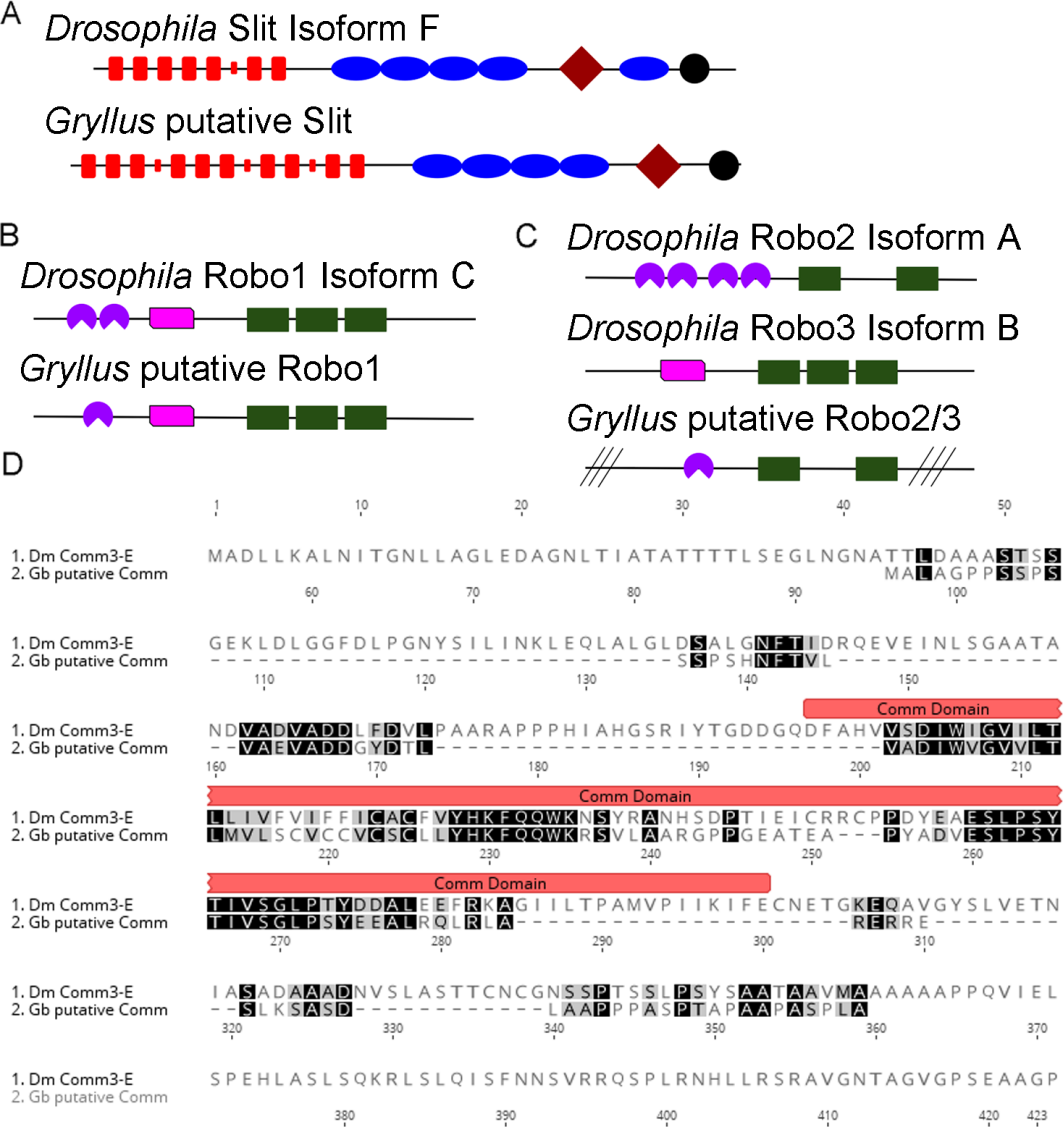
Components of the Slit signaling family are conserved in *G*. *bimaculatus*. A) The type, number, and order of protein domains was assessed by using *Drosophila* Slit, isoform F (AGB93549) and the putative *G*. *bimaculatus* Slit (TRINITY_DN152282_c0_g1_i2) as queries in a conserved domain search in NCBI. Both *Drosophila* and *G*. *bimaculatus* Slit had multiple leucine-rich repeats (red rectangles), though the number differed. Smaller red rectangles represent the C-terminal domain of a LRR. *Drosophila* Slit has five EGF domains (blue ovals), while the predicted *G*. *bimaculatus* Slit has only four. Both have a laminin G-like domain (diamond), and a C-terminal cysteine knot (black circle). B) An NCBI conserved domain search was used to identify the domains present in *Drosophila* Robo1, isoform C (AAM71113) and putative *G*. *bimaculatus* Robo1 (TRINITY_DN172482_c0_g4_i2), which included one or two IG domains (purple circular sectors) and an Ig I-set domain (pink). In addition, both predicated Robo1 proteins had three Fibronectin-type 3 domains (green rectangles). C) An NCBI conserved domain search was used to identify the domains present in *Drosophila* Robo2, isoform A (AAF51375) and Robo3, isoform B (AGB92403) and putative *G*. *bimaculatus* Robo2/3 (TRINITY_DN174950_c3_g2_i1). Both *Drosophila* Robos had several Ig domains predicted, but only Robo 2 showed specific hits in the conserved domain search (purple). *Drosophila* Robo3, isoform A possessed a single Ig I-set domain. These Robos had either three or two fibronectin-type 3 domains (green rectangles). The predicted *G*. *bimaculatus* Robo2/3 has characteristics that matched Robo 2 better (at least one Ig domain), though the overall sequence similarity was closer to Robo 3, isoform B. D) Alignment of Commisureless from *G*. *bimaculatus* (Gb putative Comm) and *Drosophila melanogaster* (Dm Comm3-E). Black shading indicates identical amino acids, gray shading indicates amino acids. The *Drosophila* comm domain is noted by a red bar above the sequence.

The Slit ligand is one of the main guidance factors that influences axonal [37] and dendritic [26] guidance during CNS development. Slit is a large extracellular matrix protein released by midline glia [38]. In invertebrates, Slits contain multiple leucine-rich repeats, several EGF-repeats, a laminin-G domain, and a C-terminal cysteine knot (Fig 2A; [39]) The Slit receptor, known as Roundabout, or Robo, was first discovered in a *Drosophila* mutagenesis screen designed to identify factors that influence axon guidance about the midline [40]. Robos typically possess multiple Ig-like domains and two or three fibronectin domains, in addition to conserved cytoplasmic motifs (Fig 2B and 2C). *Drosophila* express three different Robos Robo1, 2 and 3; Robo2 and Robo3 are thought to be the result of a recent duplication event in Diptera [41]. Most insects analyzed appear to have only two Robo receptors, Robo1 and Robo2/3. These in turn were likely a result of an earlier duplication event, since *C*. *elegans* only express a single Robo [41]. Growth cones that express Robo are unable to grow towards the Slit-expressing midline. When a growing axon must cross the midline, it first lowers Robo protein levels, grows across the midline, and subsequently re-expresses Robo on the growth cone, which facilitates movement away from the midline and prevents the growth cone from getting stuck crossing the midline repeatedly. In *Drosophila*, a protein called Commisureless (Comm) is responsible for reducing Robo expression in the growth cone tip at the proper time, which then allows that axon to cross the midline [42]. While *comm* has been found in several dipteran species, it is not thought to be widely conserved [43].

Searches of the *G*. *bimaculatus* transcriptome using *D*. *melanogaster* isoforms of Slit, Robo (1, 2 and 3) and Comm identified transcripts encoding putative homologs of each of these protein families. Specifically, the search of our *G*. *bimaculatus* transcriptome for Slit-encoding transcripts returned four hits with E-values of 0.0. Translation of the top hit, TRINITY_DN152282_c0_g1_i2, revealed a 1,459-amino acid full-length protein (Fig 2A), that was identified as most similar to slit, isoform F (**Accession No.** **AGB93549**) when the sequence was used to query the *D*. *melanogaster* proteins in FlyBase. A conserved domain search indicated that the predicted protein possessed all of the anticipated domains, though there were some minor differences in number and relative location when compared to its *Drosophila* homologue (Fig 2A). For example, *Drosophila* Slit, isoform F, has seven full-length leucine rich repeats (LRRs), and a single LRR C-terminal domain. The *G*. *bimaculatus* protein is predicted to have 10 LRRs and 3 LRR C-terminal domain regions. The predicted *G*. *bimaculatus* protein is also missing the EGF domain that is found between the laminin G-like domain and the C-terminal cysteine knot (Fig 2A). Using PCR, we have amplified and sequenced ~3,400 base pairs of the open reading frame of the *G*. *bimaculatus slit* transcript, as well as a portion of the 3’ UTR. Our confirmed sequence begins 984 base pairs into the open reading frame and contains three internal gaps of a predicted 311 base pairs. Within the confirmed sequence, we find a single difference at base 2041 of the open reading frame. A substitution of a thymine for an adenine changes the predicted amino acid from an asparagine to a tyrosine (S2 Fig). Repeating this sequencing on additional animals would be needed to confirm this difference.

The search for *G*. *bimaculatus* Robo encoding transcripts also returned numerous hits with low E-values, specifically five with E-values of 0.0 for Robo1 and two with E-values of 0.0 for Robo3. Results identical to the Robo3 search were obtained when searching the transcriptome using Robo2 as a query. Translation of the top hit from the Robo1 search, TRINITY_DN172482_c0_g4_i2, revealed a 1,602-amino acid full-length protein (Fig 2B), which was found to be most similar to Robo1, isoform C (**Accession No.** **AAM71113**), when it was used to query the *D*. *melanogaster* proteins in FlyBase. The putative *G*. *bimaculatus* Robo1 was 40.2% identical to the *Drosophila* Robo1 isoform (Table 4). Translation of the top hit identified in both the Robo2 and 3 search, TRINITY_DN174950_c3_g2_i1, revealed a 1,340-amino acid internal fragment (Fig 2C) that was identified as most similar to *D*. *melanogaster* Robo3, isoform B (**Accession No.** **AGB92403****)**, and was 40.3% identical to its *Drosophila* orthologue (Table 4). Two full-length Robo1 transcripts (TRINITY_DN172482_c0_g4_i4 and TRINITY_DN172482_c0_g4_i2) were also found, which varied from each other only due to a 30-amino acid insertion from positions 906–935 in TRINITY_DN172482_c0_g4_i4. Using one of the full length Robo1s (TRINITY_DN172482_c0_g4_i2) as a query in a conserved domain search revealed only a single Ig domain in the predicted *G*. *bimaculatus* protein instead of two as found in *Drosophila*. The remainder of the predicted protein was identical to the *Drosophila* ortholog, possessing a single Ig I set domain and three fibronectin type 3 domains (Fig 2B). Our top Robo2/3 hit in *G*. *bimaculatus* had domains identical in type when compared with *Drosophila* Robo 2; both possessed at least one Ig domain and two fibronectin type 3 domains but not the Ig I set domain found in *Drosophila* Robo 3. Though the predicted *G*. *bimaculatus* protein had three fewer Ig domains than *Drosophila* Robo 2, the *G bimaculatus* sequence was incomplete, missing both the C and N-terminal portion of the protein (Fig 2C). Analysis of the entire coding sequence would be needed to determine the number of Ig domains. Though the domain type and structure were more similar to *Drosophila* Robo2, the overall sequence was more similar to *Drosophila* Robo3 (40.3% identity, Table 4). Using PCR, we have amplified and sequenced 3,075 bp of the 4,806 bp predicted open reading fame of the putative *G*. *bimaculatus* Robo1. Our confirmed sequence begins 909 base pairs into the ORF, ends 33 bp from the stop codon, and contains two internal gaps (S2 Fig). The 3,075 confirmed bases are identical to those predicted from the transcriptome.

Searching the *G*. *bimaculatus* transcriptome for putative Comm-encoding transcripts identified four hits, all with high E-values (0.088 or higher) and from the same Trinity grouping, *i*.*e*., TRINITY_DN156272_c0_g2_X. Translation of the top hit from the Comm search, TRINITY_DN156272_c0_g2_i3, revealed a 145-amino acid internal fragment protein whose most similar counterpart in the annotated *D*. *melanogaster* dataset is Comm3, isoform E (**Accession No.** **AHN58097**). Using this putative Comm as a query in a conserved domain search revealed a single comm superfamily domain from amino acid 30–111. Alignment of the protein deduced from our transcriptome (TRINITY_DN156272_c0_g2_i3, Gb putative Comm) and *Drosophila* Comm (Dm-Comm3-E) is shown in Fig 2D. Though the overall identity between these two proteins is low (25%), an 83-base pair portion of the putative comm domain shows a nearly 50% identity with that of Dm-Comm3E.

The presence of Slit, at least two Robos, and a Comm-like protein suggests that these proteins are playing some role in the adult prothoracic ganglion, either in control or deafferented crickets. The identification of only two Robos is predicted based on work in other insect orders [41]. The presence of a Comm-like protein is a bit surprising, since it has not been well-described outside of Diptera [43]. For example, no *comm* orthologues have been identified in *Tribolium* and the introduction of *Drosophila comm* into *Tribolium* does not affect the levels of Robo [41], as would be expected based on its function in Diptera [42]. Using *Drosophila* Comm3, isoform E (**Accession No.** **AHN58097**) as a query in a tblastn search in which Diptera were excluded returned 29 hits, all with relatively high E-values. The top hit (6e-11) was a predicted, uncharacterized protein in *Agrilus panipennis* (emerald ash borer; **Accession No.** **XM_018464809**). Aligning the Comm-like proteins from *Agrilus* and *G*. *bimaculatus* reveals an overall identity of nearly 40%, but a 65% identity within the putative comm domain (data not shown).

The functions that Slit, Robo, and Comm play in the adult cricket prothoracic ganglion remain to be determined. In developing *Drosophila*, Slit is released by midline glial cells [37] and through its diffusion, sets up a gradient that decreases in concentration from the midline [27]. Axon growth cones that express Robo are repelled from the midline. In addition to midline guidance, Slit-Robo signaling appears to help create three longitudinal axonal pathways in the developing CNS of many insects [44]. Mutation analysis indicates that these different functions, midline repulsion or lateral positioning, are dependent on specific Ig-like domains of Robo [45].

The expression of Slit and Robo are known to persist in the adult rodent peripheral nervous system (PNS) and CNS [46,47], and a subset of Slits and Robos are upregulated after lesions or ischemia in rodent CNS [48], suggesting that this signaling pathway may play a role in injury repair. Further expression and functional analysis will need to be completed in the cricket to understand the roles of Comm, Slit, and Robo in the developing and adult nervous system of this insect. Given the role that the Slit-Robo signaling system plays at the midline in *Drosophila*, one could hypothesize that a downregulation of Robo in deafferented AN neurons would allow those dendrites to cross the midline, though presumably additional guidance molecules would be needed to mediate the growth, branching, and synapse formation that occurs subsequently [12,49].

### Netrin signaling

**Fig 3:**
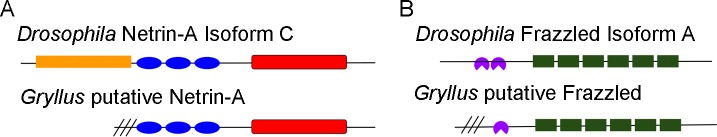
Netrin and Frazzled proteins in *Drosophila* and *G*. *bimaculatus* share most domains. A) The type, number, and order of protein domains was assessed by using *Drosophila* Netrin A, isoform C (AHN59713) and the putative *G*. *bimaculatus* Netrin A (TRINITY_DN160784_c2_g1_i2) as queries in a conserved domain search. *Drosophila* Netrin A, isoform C possessed a laminin N-terminal domain (yellow rectangle), three laminin-type EGF-like domains (blue ovals), and a netrin-like domain (red rectangle). The angled lines on the *G*. *bimaculatus* protein representation indicate missing N-terminal sequence, but the remaining sequence matches that of *Drosophila*, with three laminin-type EGF-like domains (blue ovals), and a netrin-like domain (red rectangle). B) The type, number and order of protein domains in *Drosophila* Frazzled, isoform A (AAF58493) and the putative *G*. *bimaculatus* Frazzled (TRINITY_DN172587_c0_g1_i7) was assessed using a conserved domain search. Frazzled had at-least two Ig-like domains (purple) and 6 Fibronectin Type III domains. The predicted *G*. *bimaculatus* protein was similar, though some sequence was missing from the N-terminal end (angled lines).

Netrins were first discovered in vertebrates in a screen designed to identify the floor plate chemoattractant that is important for guiding commissural axons in the developing spinal cord [50]. Subsequent work identified two homologous proteins in *Drosophila*, Netrin-A and Netrin-B [32,51]. Netrins are related to the laminin superfamily of proteins and contain a 600-amino acid N-terminal laminin-like domain, three epidermal growth factor (EGF) repeats, and a netrin-like domain (Fig 3A). Netrins bind to at least three main families of receptors, the deleted in colorectal cancer (DCC) family [52], the Unc-5 family [53], and down’s syndrome cell adhesion molecule [54]. Orthologs of each of these protein families are found in *Drosophila*; Frazzled, the DCC family member found in *Drosophila*, mediates attraction, [31], *Drosophila* Unc-5 (Dunc-5) mediates repulsion [55], and Dscam likely acts as a co-receptor. Dscam actions are complex due to alternative splicing, which can create over 38,000 different isoforms in *Drosophila* [56]. Frazzled is a single-pass transmembrane protein that has C2-type Ig domains and fibronectin type III repeats (Fig 3; [31]). The cytoplasmic tail has three P-motifs, which are likely important for dimerization as well as interacting with downstream signaling molecules [57]. *Drosophila* Unc5 was found to be expressed in a subset of axons that exit the CNS without crossing the midline [55], and characterization of the receptor shows it to contain two Ig domains and two thrombospondin type 1 domains as well as intracellular domains important for interacting with DCC [58].

Searches of the *G*. *bimaculatus* transcriptome using components of the Netrin signaling pathway identified transcripts encoding putative homologs of Netrin-A, Frazzled, Unc-5 and Dscam. While both Netrin-A and Netrin-B were searched for in the *G*. *bimaculatus* dataset, the top hits (two with E-values of e-119 or higher) returned in each search were identical and had lower E-values when the former protein was used as the BLAST query. Translation of the top hit for both searches, TRINITY_DN160784_c2_g1_i3, revealed a 340-amino acid C-terminal partial protein (Fig 3A), which when used to query the extant *D*. *melanogaster* protein dataset in FlyBase, returned Netrin-A, isoform C (**Accession No**. **AHN59713**) as the most similar *Drosophila* protein. Aligning these two proteins indicated a 48.3% identity at the amino acid level (Table 4). Using the Netrin identified and predicted from the *G*. *bimaculatus* transcriptome as a query in a conserved domain search we found the partial predicted protein contained three EGF repeats and a netrin-like domain, though it was missing the N-terminal laminin domain (Fig 3A).

The BLAST search for *frazzled*-encoding transcripts returned five hits with E-values of 0.0, and three others with E-values of e-178 or lower. Translation of the top hit, TRINITY_DN172587_c0_g1_i7, revealed a 1,331-amino acid full-length protein (Fig 3B) that was found to be most similar to Frazzled, isoform A (**Accession No.** **AAF58493**) when it was used to query the extant *D*. *melanogaster* proteins curated in FlyBase. Alignments of these two proteins showed that 40.7% of the amino acids were identical. Alignments of the remaining, full-length proteins predict the existence of five different isoforms. Using TRINITY_DN172587_c0_g1_i7 as a query in a conserved domain search identified a predicted protein with one Ig domain, and six fibronectin-type III domains (Fig 3B). One of the longer deduced proteins (TRINITY_DN172587_c0_g1_i5) is predicted to have the expected four Ig domains, along with the expected number of fibronectin domains.

Searches for Unc-5 returned seven hits with E-values of e-62 or lower. Translation of the top hit, TRINITY_DN149614_c0_g1_i1, revealed a 254-amino acid internal fragment protein that was identified as most similar to *D*. *melanogaster* Unc-5, isoform B (**Accession No.** **AHN56248**). Aligning these two proteins showed that 37.1% of the amino acids were identical (Table 4), though the amino acids are 50% identical when limited to a well-conserved region of 212 amino acids. Four additional C-terminal transcripts in two different Trinity groups were returned as encoding putative Uncs, and aligning the predicted proteins indicates they may represent different isoforms, though evaluating more complete sequences will be necessary to make this determination.

Forty-seven hits with E-values of 0.0 were returned in the search for Dscam-encoding transcripts. An additional 52 hits had E-values lower than e-101. Translation of the top hit, TRINITY_DN173597_c2_g22_i1, revealed a 1,251-amino acid C-terminal partial protein; the most similar *D*. *melanogaster* sequence to this predicted *G*. *bimaculatus* protein was identified as Dscam1, isoform Y (**Accession No.** **ABI31042**), and these proteins are 66.8% identical in amino acid sequence to one another (Table 4). Over 20 different Trinity groups were represented in these top hits (0.0), indicating the possibility of high complexity for the Dscam family in *G*. *bimaculatus*.

The presence of predicted homologues to Frazzled, Unc-5, Dscam, and at least one Netrin indicate that Netrin signaling could play a role in normal or deafferented cricket prothoracic ganglia. Multiple copies of Netrins have been identified in several mosquito species [59], though only two Netrins, A and B, exist in *Drosophila*. In *Drosophila*, *netrin* is expressed at the midline and attracts the appropriate axons towards the midline in order to cross [51]. Functional studies in other insects and arthropods indicate that Netrin likely mediates midline attraction, but that the specific timing and regulation of this attraction differs from that in *Drosophila* [59–61]. The fact that the midline has the ability to both attract and repel axons with Netrin and Slit, respectively, means that the appropriate guidance of any given axon must involve complex spatiotemporal regulation of the expression of different receptors, a process that is not fully understood. The interplay between the attraction of the Netrin pathway and the repulsion of the Slit-Robo pathway is complex in *Drosophila*, and likely involves a number of co-receptors and other factors. Netrin itself can also repulse growth cones in the short range by binding to Unc-5, or over longer distances, by binding heterodimers of Unc-5 and Frazzled [55]. In addition, Netrin signaling may be further modified in individual cells to cooperate with additional proteins [62,63] or to regulate distinct axonal projections [64].

While the main function of Frazzled appears to be mediating attraction to Netrin, it also acts in a Netrin-independent manner to upregulate the expression of Comm, thus decreasing the ability of slit to repel Comm-expressing axons [65]. Recent evidence indicates that Frazzled can be cleaved by γ–secretase, releasing the intracellular domain to travel to the nucleus where it acts as a transcription factor to upregulate *comm*, leading to the reduction of Robo, and axon attraction to the midline rather than repulsion from it [66]. Dscam function is complex as well; recent work indicates that Dscam can interact with other receptors, such as Robo, and modify the output in response to ligands [67], making this receptor highly flexible and multi-functional during development. In addition, the potential production of dozens of differing proteins produced from alternative splicing appears to influence neural connectivity [68] and dendritic avoidance [69] during development. The potential number of alternatively spliced transcripts is consistent with the large number of Dscam hits (at least 99) identified in our transcriptome.

Netrin, Unc-5, and DCC are expressed in adult mammals [70–72]. DCC is expressed in the spines of hippocampal cells and is important for long-term potentiation in the forebrain [72]. Netrin expression has been found to be particularly high in neurogenic regions of the brain, such as the olfactory bulb, the subventricular zone, and specific regions of the hippocampus [73]. Interestingly, Unc-5 is upregulated in deafferented granule cells in the cerebellum of rats in what may be a recapitulation of a developmental state [74]. Similarly, Netrin and DCC are upregulated after ischemia in the brain of rats, and it is thought that the temporal coordination of expression facilitates axon outgrowth after injury [75]. After sensory loss in the cricket, one might expect coordinated changes in Robo, Frazzled, Dscam, and Unc-5 expression in deafferented AN dendrites, which could facilitate the growth of deafferented dendrites across the midline.

### Ephrin signaling

**Fig 4:**
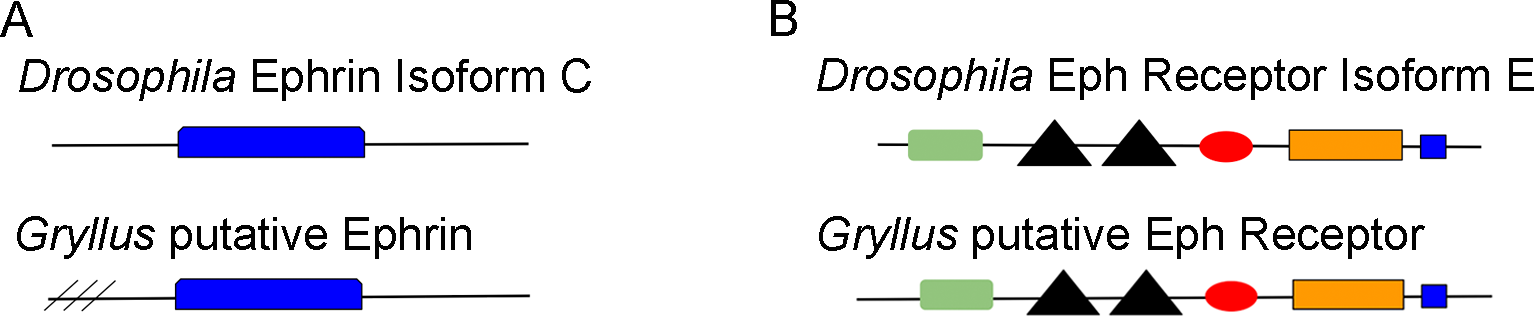
Ephrin and Eph proteins in *Drosophila* and those predicted in *G*. *bimaculatus* are identical in domain type, number and organization. The type, number, and order of protein domains was assessed by using *Drosophila* Ephrin Isoform C (AAN06500) and the putative *G*. *bimaculatus* Ephrin (TRINITY_DN159904_c0_g1_i6) as queries in a conserved domain search. In both proteins, an Ephrin domain was present (blue rectangle). Angled lines indicate incomplete *G*. *bimaculatus* sequence at the N-terminal end. B) The type, number, and order of protein domains in Drosophila Eph (AAN06503) and putative *G*. *bimaculatus* Eph (TRINITY_DN159184_c0_g1_i3) was determined. Both proteins showed identical number, order, and type of domain, which included an eph receptor ligand binding domain (green rectangle), two fibronectin type 3 domains (triangles), an ephrin type A receptor 2 transmembrane domain (red oval), an ephrin receptor protein tyrosine kinase domain (orange rectangle), and a SAM domain (blue square).

The Eph receptor family is the largest family of receptor tyrosine kinases. Discovered in a screen for oncogenic tyrosine kinases, they were named Eph for the erythropoietin-producing hepatocellular carcinoma cell line from which they were isolated. In vertebrates, the family totals 14 receptors and is divided into two subclasses, EphAs and EphBs (reviewed in [76]). Activation of these receptors by Eph receptor interacting proteins (Ephrins) can influence a wide variety of cellular processes including axon guidance, boundary formation, wound healing, and tissue maintenance [76]. *Drosophila* have a single Ephrin, which has a low overall homology to vertebrate Ephrins, but contains some regions of higher homology (Fig 4A; [77,78]).

A single Eph receptor has been found in *Drosophila*. It shows similarity to both subclass A and B Ephs, so cannot easily be placed in either class [79]. Originally named Dek, for *Drosophila* Eph Kinase, it contains all of the domains found in vertebrate Ephs, including an eph receptor ligand binding domain, two fibronectin-type III repeats in the extracellular domain, an intracellular portion containing a conserved catalytic domain, and a sterile alpha motif (SAM; Fig 4B). The SAM is thought to be important for binding SH-2 containing proteins, and the juxtamembrane region has conserved tyrosines important for the function of the catalytic domain [78,79].

Transcripts encoding both Ephrin and its receptor tyrosine kinase, Eph, appear to be present in the *G*. *bimaculatus* transcriptome. Five transcripts with E-values of e-70 or lower were returned for the Ephrin search, with translation of the top hit, TRINITY_DN159904_c0_g1_i6, revealing a 307-amino acid C-terminal partial protein; an isoform of Ephrin, isoform C (**Accession No.** **AAN06500**) was confirmed as the most similar *D*. *melanogaster* protein to this *G*. *bimaculatus* sequence. These two proteins show an overall amino acid identity of 36.1% (Table 4), though this identity is much higher (76%) when focusing on a central region of 174 amino acids (data not shown). Two additional transcripts from the same grouping were returned that predict 271-amino acid, full-length proteins when translated. These are likely different isoforms from the C-terminal partial proteins predicted by the top hit, though they both match the same *Drosophila* Ephrin isoform. A conserved domain search for both the *G*. *bimaculatus* and *Drosophila* Ephrin show a single ephrin ectodomain (Fig 4A).

Eight transcripts with E-values of 0.0 were returned for the Eph search. Translation of the top hit from this search, TRINITY_DN159184_c0_g1_i3, revealed a 996-amino acid full-length protein; the most similar *D*. *melanogaster* protein to this *G*. *bimaculatus* sequence was found to be Eph receptor tyrosine kinase, isoform E (**Accession No.** **AAN06503**). These two proteins show a 59.5% identity at the amino acid level (Table 4). The remaining seven transcripts also are predicted to encode full-length proteins, with four isoforms evident. A conserved domain search of the *G*. *bimaculatus* Eph showed similarity in type, number, and order of domains when compared to the *Drosophila* homolog, including an Eph receptor ligand binding domain, two fibronectin type 3 domains, and Ephrin type A receptor 2 transmembrane domain, an ephrin receptor protein tyrosine kinase domain, and a SAM domain (Fig 4B).

The Eph receptor and ligand appear to coordinate multiple aspects of nervous tissue development in *Drosophila*. Within the developing CNS, Ephrin is expressed outside axonal tract areas, such as on the edges of the CNS, while the axons themselves express the Eph receptor. This complementary expression is thought to coordinate with Slit-Robo signaling to funnel the axons into distinct tracts, both longitudinal and commissural, and it prevents the abnormal exiting of axons from the CNS [77]. In vertebrates, an Eph gradient in the developing visual system is important for topographic map formation, demonstrating a conservation of function for Eph-Ephrin signaling and topographic map formation across the animal kingdom [78].

Since Ephrins are membrane-associated, any guidance of axons is due to cell-cell contact. In addition to traditional ligand-receptor signaling, or forward signaling, Eph receptors can induce reverse signaling through membrane-associated Ephrins [76]. This bidirectional Eph-Ephrin signaling creates an important role for them in synapse formation and plasticity [80], given that contact between axons and dendrites will be conveyed bidirectionally to both cells. The combined ability to guide axons to a stopping point and to initiate synapse formation is evident in a number of developing tissues where gradients of Ephs and Ephrins are used to set up topographic maps [81,82].

The presence of Eph and Ephrin in the adult cricket is not surprising, given that expression of this ligand-receptor pair is not limited to development in other animals. Typically, the adult mammalian expression is lower and more limited spatially than in development (reviewed in [83]), and further studies would need to be completed in adult and developing crickets to understand the expression differences over time. Injury typically leads to the upregulation of Eph and Ephrin expression in mammals, and it is thought that this upregulation may be one of the factors that actively inhibits regeneration in the CNS (reviewed in [83]). It will be interesting to determine whether Ephrin and Eph are differentially regulated after injury in the cricket and in which direction. Perhaps one reason the AN dendrites in the prothoracic ganglion are capable of such robust anatomical plasticity is due to the downregulation of inhibitory signaling pathways, such as the Eph-Ephrins. In addition, one could imagine Eph-Ephrin signaling being important for targeting growing dendrites to the appropriate region, identifying the point at which they should stop growing, and aiding in the formation of contralateral synapses. Previous work has shown a sexual dimorphism in male and female dendritic growth after deafferentation [13], with female dendrites growing only half as far as male dendrites. If Ephrin signaling were involved in stopping dendritic growth and facilitating synapse formation, one might predict a sexual dimorphism in Eph/Ephrin signaling after deafferentation.

### Semaphorin signaling

**Fig 5:**
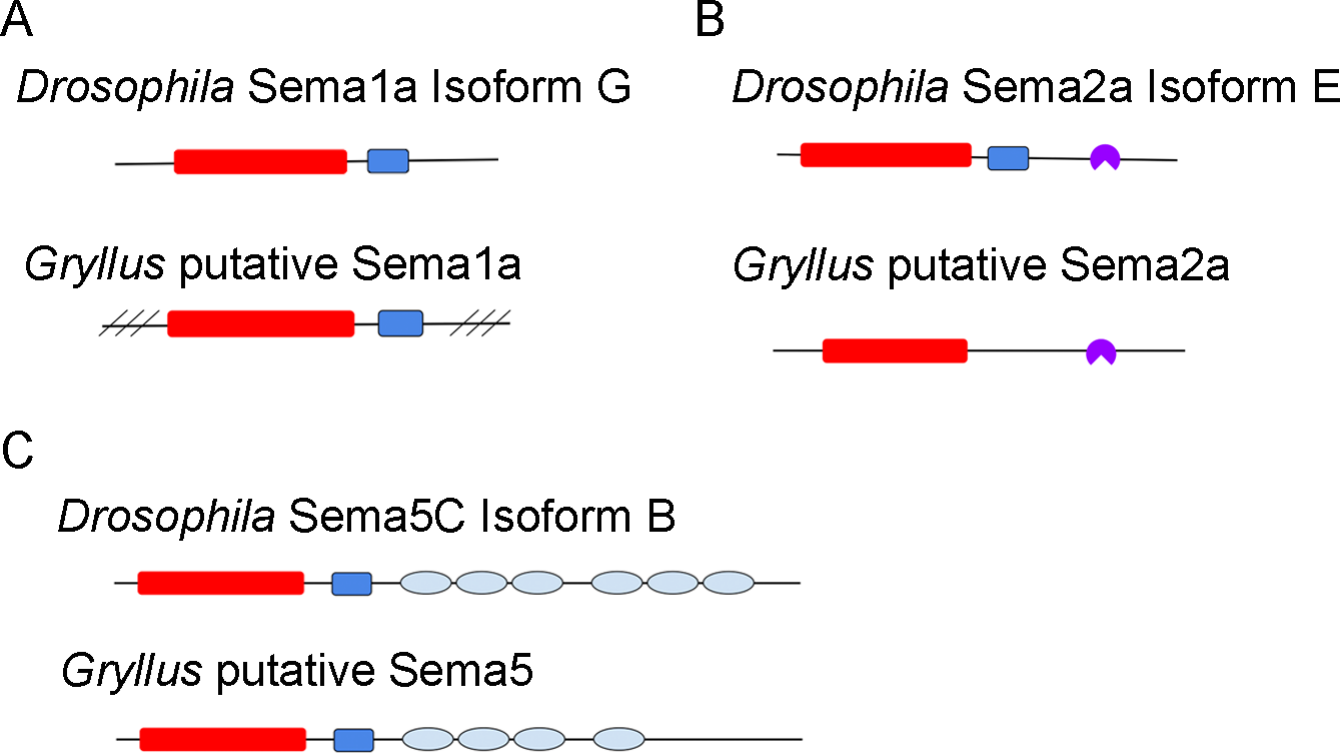
Semaphorin proteins predicted from the *G*. *bimaculatus* transcriptome share most of the functional domains found in *Drosophila* Sema proteins. A) The type, number, and order of protein domains was assessed using *Drosophila* Sema1a, isoform G (AGB92801) and the putative *G*. *bimaculatus* Sema1a (TRINITY_DN169697_c2_g1_i3) as queries in a conserved domain search. Both proteins possessed a sema domain (red rectangle) and a plexin repeat (blue rectangle). Diagonal lines indicate sequence missing from the N- and C-terminal ends of the predicted *G*. *bimaculatus* protein. B) The type, number, and order of protein domains was assessed using *Drosophila* Sema2a, isoform E (AAS64837) and the putative *G*. *bimaculatus* Sema2a (TRINITY_DN173773_c2_g2_i6) as queries in a conserved domain search. Both proteins possessed a sema domain (red rectangle) and an Ig domain (purple). *Drosophila* Sema2a, isoform E possessed a plexin repeat (blue rectangle) which was missing in *G*. *bimaculatus*. C) The type, number, and order of protein domains was assessed using *Drosophila* Sema5C, isoform B (ACZ94701) and the putative *G*. *bimaculatus* Sema5 (TRINITY_DN165105_c1_g2_i8) as queries in a conserved domain search. Both proteins possessed a sema domain (red rectangle) and a plexin repeat (blue rectangle). *Drosophila* Sema5c isoform B had six thrombospondin type 1 domains while the predicted Sema5 in *G*. *bimaculatus* had just four (light blue ovals).

The Semaphorin (Sema) family is a large family of proteins that influences a wide-range of processes. The first Sema was originally discovered as an axon guidance factor in embryonic grasshopper [84]. The members of this family are now grouped into eight different classes. Invertebrates express transmembrane (class 1 and class 5) and secreted forms (class 2), and vertebrate Semas are similarly organized into classes based on structure (reviewed in [85]). An 8^th^ class is expressed in viruses [86]. All members of the Sema family possess a well-conserved Semaphorin domain that is approximately 500 amino acids long and contains between 14–16 cysteine residues. Individual Semas can be distinguished by the presence of additional domains, such as a “plexin, semaphorin, integrin” (PSI) domain, Ig-like domains, or thrombospondin domains (Fig 5; reviewed in [86]).

Semas mainly bind Plexin receptors in invertebrates, though they also interact with a variety of additional co-receptors. Sema1a and 1b bind Plexin A [87], while Sema2a binds Plexin B [88]. Plexins themselves contain a sema domain and are thought to be ancestrally related to Semas [87]. In invertebrates, co-receptors include Off-Track, a putative tyrosine kinase receptor which associates with Plexin A [89], and Guanylyl cyclase at 76C, which has a catalytic cyclase domain. This latter co-receptor is presumed to induce local increases in cGMP that are critical for repulsion as a result of Sema signaling [90].

Transcripts encoding putative homologs of Sema1, Sema2 and Sema5, Plexin A, Plexin B, Off-Track and Guanylyl Cyclase at 76C were identified in the *G*. *bimaculatus* transcriptome. Sema1 searches were conducted using *D*. *melanogaster* isoforms of both Sema1a and Sema1b. The *G*. *bimaculatus* hits returned for both queries were essentially identical, with lower E-values seen for the Sema1a query, i.e. 13 with E-values of 0.0 vs. e-142 being the E-value for the top Sema1b query. Translation of the top hit for Sema1a,TRINITY_DN169697_c2_g1_i3, revealed a 782-amino acid internal partial protein (Fig 5A), a protein identified as most similar to Sema1a, isoform G (**Accession No.** **AGB92801**) when it was used to query the *Drosophila* proteins in FlyBase. These proteins are 59.5% identical at the amino acid level (Table 4). Similarly, the Sema2 searches were conducted using isoforms of both *D*. *melanogaster* Sema2a and Sema2b as queries, with the top hits returned for both being essentially identical. Translation of the top hit from both the Sema2a and the Sema2b search, TRINITY_DN173773_c2_g2_i6, revealed a 726-amino acid full-length predicted protein (Fig 5B), which was identified as most similar to Sema2a, isoform E (**Accession No.** **AAS64837**) when it was used to query the *D*. *melanogaster* proteins in FlyBase. These two proteins, when aligned, showed a nearly 70% identity at the amino acid level. Within the six hits that matched Sema2a, at least two isoforms were evident. Using these top *Drosophila* and *G*. *bimaculatus* Sema1a and 2a hits as queries in a conserved domain search through NCBI, we compared protein domain type, number, and relative order and found a high level of domain similarity in these proteins, though the *G*. *bimaculatus* Sema2a was missing a plexin repeat domain (Fig 5A, B). We have independently amplified and sequenced the portions of the predicted open reading frames of both *Sema1a* and *Sema2a*, verifying the sequences presented here. Sanger sequencing of 2,351 bases of the *G*. *bimaculatus sema1* ORF (**Accession No.** **MF817714**) found different bases at 15 locations (see S2 Fig). The majority of these base pair differences did not change the predicted amino acid at that location, though we did find that our sequencing predicted the addition of a single glutamine at position at amino acid 362. The *sema2a* ORF predicted from our top RNA-Seq hit is 2,181 bp long, and 1,303 of these bases have been confirmed through sequencing (**Accession No.** **ABK35089**). Though there were four individual bases that differed from that predicted from our RNA-Seq, the predicted protein is identical (S2D Fig).

Five hits with E-values of 0.0 were returned when the *G*. *bimaculatus* transcriptome was searched for putative Sema5-encoding transcripts. Translation of the top hit from this search, TRINITY_DN165105_c1_g2_i8, revealed a 1,057-amino acid full-length protein whose most similar counterpart in the annotated *D*. *melanogaster* dataset was found to be Sema5c, isoform B (**Accession No.** **ACZ94701**). At least two additional isoforms were evident in the remaining four transcripts. An NCBI, conserved domain analysis of this full-length *G*. *bimaculatus* protein revealed a domain structure similar to *Drosophila* Sema5c, isoform A, including a Sema domain, and a plexin repeat domain, but only four thrombospondin type I repeats instead of the six predicted from *Drosophila* (Fig 5C).

**Fig 6:**
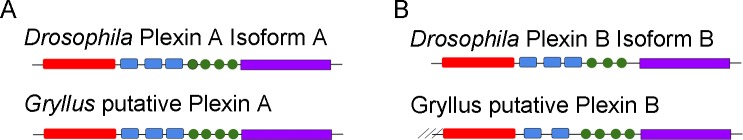
Plexin proteins predicted from the *G*. *bimaculatus* transcriptome share most of the functional domains found in *Drosophila* Plexin proteins. A) The type, number, and order of protein domains was assessed using *Drosophila* Plexin A, isoform A (AAF59394) and the putative *G*. *bimaculatus* Plexin A (TRINITY_DN159247_c0_g1_i2) as queries in a conserved domain search. Both proteins possessed a sema domain (red rectangle), three plexin repeats (blue rectangle), four IPT domains (Ig, Plexin Transcription factor domains; green circles), and a rasGAP domain (purple rectangle). B) The type, number, and order of protein domains was assessed by using *Drosophila* Plexin B, isoform B (AFH06760) and the putative *G*. *bimaculatus* Plexin B (TRINITY_DN161683_c0_g1_i2) as queries in a conserved domain search. Both proteins possessed a Sema domain (red rectangle) two or three plexin repeats (blue rectangles), three or four IPT domains (Ig, Plexin Transcription factor domains; green circles), and a rasGAP domain (purple rectangle). Diagonal lines indicate sequence missing from the N-terminal end of the *G*. *bimaculatus* protein.

Ten hits with E-values of 0.0 were returned when the *G*. *bimaculatus* transcriptome was searched for Plexin A encoding transcripts, while 11 hits with E-values of 0.0 were returned for the search for Plexin B-encoding sequences. Translation of the top hit from the Plexin A search, TRINITY_DN159247_c0_g1_i2, revealed a 1,909-amino acid full-length protein (Fig 6A), whose most similar *Drosophila* counterpart was found to be Plexin A, isoform A (**Accession No.** **AAF59394**). Alignments of these two proteins indicate that they are nearly 70% identical at the amino acid level (Table 4). Of the eight, full-length transcripts identified within the top hits, five isoforms were evident. A conserved domain search of both the top *G*. *bimaculatus* hit and its *Drosophila* Plexin A counterpart revealed identical protein domains in predictable order, including a sema domain, three plexin repeat domains, four domains known as “immunoglobulin-like fold, Plexins, Transcription factors” (IPTs), and a rasGAP domain (Fig 6A). Translation of the top hit from the Plexin B search, TRINITY_DN161683_c0_g1_i2, revealed an 1,850-amino acid C-terminal full-length protein (Fig 6B) that was identified as most similar to *D*. *melanogaster* Plexin B, isoform B (**Accession No.** **AFH06760**). When aligned, these two proteins showed a 60.1% identity. Using this predicted *G*. *bimaculatus* protein and the top *Drosophila* hit as queries in a conserved domain search, we found highly similar proteins that differed slightly in domain number; the *G*. *bimaculatus* Plexin B had only two plexin repeats instead of the expected three, but had four IPT domains instead of the expected three (Fig 6B). Using PCR, we have independently amplified and sequenced a portion of the 5,553 bp predicted open reading frame of *PlexB*. Our confirmed sequence starts 1,918 bases from the beginning of the ORF and contains two gaps totaling 1,092 bases (S2E Fig). The 2,544 bases that were successfully sequenced do not differ from the sequence predicted from our RNA-Seq assembly.

Querying the *G*. *bimaculatus* transcriptome for co-receptors identified two putative proteins. The search for Off-Track-encoding transcripts returned seven hits, all from the same Trinity grouping, *i*.*e*., TRINITY_DN160729_c0_g1_X, with E-values of e-72 or lower. Translation of the top hit from this search, TRINITY_DN160729_c0_g1_i15, revealed a 680-amino acid internal partial protein, whose sequence was found to be most similar to Off-Track, isoform A (**Accession No.** **AAF58596**) when it was used to query the annotated *D*. *melanogaster* proteins curated in FlyBase. Alignment of these two proteins showed a 27.2% identity at the amino acid level, though a central region of about 370 amino acids show an identity of 41%. Though no full-length proteins were recovered, at least five isoforms were evident in the sequences found.

Three hits with E-values of 0.0, from two different Trinity groupings (TRINITY_DN166112_c0_g1 and TRINITY_DN162371_c0_g1) were returned when the *G*. *bimaculatus* transcriptome was searched for putative Guanylyl Cyclase at 76C-encoding transcripts. Translation of the top hit from this search, TRINITY_DN166112_c0_g1_i2, revealed a 1,135-amino acid internal protein fragment, a protein whose most similar *Drosophila* counterpart was identified as Guanylyl Cyclase at 76C, isoform D (**Accession No.** **AGB94756**). Alignments of these two proteins showed them to be 58.1% identical (Table 4).

The expression of Sema ligands and receptors persist into adulthood in crickets and other organisms [91,92], and Sema signaling has been shown to be important to proper function in adult insects and mammals [92–95]. Thus, it is not surprising to find Semas, Plexins, and two co-receptors in our adult prothoracic ganglion transcriptome. Our transcriptome did not appear to have Sema1b or Sema2b homologues. The absence of these two predicted proteins might have occurred for at least three reasons, 1) perhaps these two transcripts are not expressed in the adult, 2) these well-conserved orthologues do not exist in the cricket, or 3) perhaps they are present but are expressed at extremely low levels and did not get represented in the final transcriptome. Despite the absence of these two predicted proteins, the presence of the remaining ligands and receptors implies that Sema signaling is important in the normal or deafferented adult prothoracic ganglion.

Sema signaling during development is central to midline crossing, branching, and synapse formation. Work in a variety of organisms has shown that Semas are capable of guiding both axon and dendrites during development [24,96,97]. For example, Semas influence the positioning of major commissural projections in the mouse brain [98]. Dendritic growth, branching, and spine formation, as well as synapse formation, are also influenced by Sema signaling [99–104], indicating that they play an important role during neuronal development.

Predicting the function of Semaphorin signaling in the adult is difficult, since the role of Sema signaling in mature tissues is not well understood. Experiments in mammals indicate potential roles in newborn cells in the adult and in modulating synaptic transmission. For example, in the adult mouse hippocampus, Sema5a inhibits synaptogenesis in adult-born cells [94], while Sema3s promote dendritic growth in these cells [105]. Secreted Semas have also been shown to modulate synaptic transmission in the adult hippocampus [106], indicating that they not only influence growth and synaptogenesis, but that they also have a role to play in synaptic modulation. Semaphorin signaling ligands and receptors are also present in the adult spinal cord [107], and may play an important role in the failure of effective regeneration after injury [23,108,109]. In the cricket, Sema signaling could be important at a number of points after deafferentation, including midline crossing, dendritic growth and branching, and synapse formation.

### Intracellular sema signaling pathway components

The intracellular signaling cascade downstream of Sema binding in invertebrates is not completely understood. In *Drosophila*, Sema1 binding to Plexin A recruits the PKA anchoring protein Nervy. Nervy’s hypothesized ability to anchor a specific PKA to Plexin A may effectively couple PKA-induced protein phosphorylation to Plexin A activation in a spatiotemporal manner [110]. Activated Plexin A also interacts with Molecule interacting with CasL (Mical), an unusual protein that is capable of oxidizing nearby proteins. Mical could potentially influence the local stability of the actin cytoskeleton through oxidation [111]. Sema2 activation of Plexin B in *Drosophila* is thought to sequester the small GTPase, Rac, limiting Rac’s ability to influence the actin cytoskeleton, while Plexin B activation simultaneously activates a different small GTPase, Rho, which typically results in actin depolymerization [112]. This results in a reduction in actin extension and ultimately growth cone repulsion [113,114].

Searches of the *G*. *bimaculatus* transcriptome for sequences encoding the intracellular components of the Semaphorin signaling pathway identified transcripts putatively encoding homologs of Mical, PKA, Rac1 and Rho1. The search for Mical-encoding transcripts identified six sequences with E-values of 0.0. Translation of this top hit, TRINITY_DN175376_c0_g1_i11, revealed a 2,614-amino acid full-length protein, which was found to be most similar to Mical, isoform P (**Accession No.** **ALI30552**) when it was used to query the extant *Drosophila* proteins in FlyBase for homologous sequences. The overall alignment of these two proteins indicated only a 27.2% identity at the amino acid level (Table 4), though when the first 690 amino acids were examined, there was a 60% identity. Two transcripts with E-values of e-127 were identified from the *G*. *bimaculatus* transcriptome as encoding putative cAMP-dependent, type 2 PKA homologs (PKA-R2). Translation of the top hit from this search, TRINITY_DN165220_c0_g2_i1, revealed a 382-amino acid full-length protein whose closest *D*. *melanogaster* homolog was confirmed to be PKA-R2, isoform B (**Accession No.** **AAM71046**). Alignments of these two proteins indicated a 64.6% identity at the amino acid level. The search for Rac1-encoding transcripts returned six hits with E-values of e-104, while the search for Rho1-encoding sequences returned thirteen hits with E-values of e-103. Translation of the top hit from the Rac1 search, TRINITY_DN170604_c0_g3_i2, revealed a 192-amino acid protein that we presume to be full length, though a 3’ UTR stop codon was not evident, which could be consistent with an incomplete 3’ UTR sequence. Our assumption about the protein is bolstered by how well it aligns with its closest *Drosophila* counterpart, Rac1, isoform B (**Accession No.** **AGB93942**). These proteins were 94.8% identical overall, with a 100% identity over the first 47 amino acids (data not shown). Translation of the top Rho-1 hit, TRINITY_DN165708_c0_g2_i4, revealed a 192-amino acid full-length protein that was found to be most similar to *D*. *melanogaster* Rho1, isoform A (**Accession No.** **AAM70944**). Alignments of these two proteins showed them to be 94.3% identical at the amino acid level (Table 4).

### Potential use of our adult prothoracic ganglion transcriptome for investigating compensatory plasticity

It is important to note that the prothoracic ganglion transcriptome described here was constructed from both control and deafferented individual adult male prothoracic ganglia at three time points. The stress and impact of general injury was controlled for by removing a portion of the tarsus of control animals. At the current level of analysis, however, we have not determined whether the identified guidance proteins are present at detectable levels in the control or deafferented crickets or both. Future work with the dataset, through differential expression analysis and quantitative PCR, will seek to explore the relative changes in gene expression in response to deafferentation at each of the three time points. Previously, it was demonstrated that expression of *Sema2a* in the prothoracic ganglion is significantly increased 7 days after deafferentation compared to controls [115]. Differential expression analysis would aim to corroborate previous findings and extend the scope of analysis. Identifying which RNA transcripts show significant changes in expression (both those corresponding to proteins identified here and others not necessarily related to guidance cues) will generate candidate genes that may play a role in the molecular control of the compensatory growth process.

## Materials and methods

### Animals and tissue dissection

Mediterranean field crickets, *G*. *bimaculatus* (N = 21) from an inbred colony, originally obtained from the Hoy Lab (Cornell University, Ithaca, NY), were used as the source of prothoracic ganglionic RNA for construction of the transcriptome in which we confirmed the predicted sequences. Prothoracic ganglia from additional crickets were used for PCR and sequencing in order to confirm transcriptome-predicted sequences. Crickets were kept in a twelve-hour light and dark cycle at 40–60% humidity and 28°C with cat chow and water *ad libitum*. For the transcriptome experiment, batches of juveniles were removed from the colony bins and placed in plastic cages together with water and food *ad libitum* and egg flats for shelter. After their final molt, adult male brown-morph crickets were placed in a plastic cage with other new adults and received water and food *ad libitum* and egg flats for shelter. Three to five days following the final adult-molt, the left prothoracic limbs were amputated either on the femur one centimeter above the tympanic membrane (“deafferented” experimental condition) or at the most distal segment of the tarsus (the “foot-chop” control condition), leaving the tympanic membrane intact.

Prothoracic ganglia were removed from crickets 24 hours, 3 days, and 7 days post-deafferentation (N = 3 at each time point) or control amputation (N = 3 at each time point) for RNA purification, for a total of 18 samples. An additional three prothoracic ganglia were extracted from crickets that had been backfilled with a fluorescent neuronal tracer 24 hours earlier (see below). Prior to dissection, crickets were placed in a 4°C refrigerator and subsequently in a petri dish on ice to slow movement and facilitate dissection. Crickets were pinned ventral side up through the head and abdomen. Except for the two forelegs, all extremities were removed. The remaining forelegs were pinned to facilitate dissection of the thoracic region. The prothoracic ganglion was then removed from each cricket and placed into 500ml QIAzol Lysis Reagent (QIAGEN) and kept on ice.

### RNA isolation

RNA was purified from the 21 individual prothoracic ganglion samples using the QIAGEN RNeasy Lipid Tissue Minikit (QIAGEN). Briefly, after removal from the cricket, each ganglion was homogenized by hand with a pestle. The ganglia were either stored at -20°C or immediately put through the remainder of the RNA extraction. Additional QIAzol lysis buffer was added to bring the volume to 1 mL. After 5 minutes of incubation at room temperature, 200 μl of chloroform was added. After 15 sec shaking, samples were incubated for 2 min at RT. Samples were centrifuged at 12,000g for 15minutes at 4°C. The aqueous phase was transferred to a new tube and 500 μl of 70% ethanol was added. QIAGEN RNeasy Mini Spin Columns were used to purify RNA. RNA was eluted from the column with 30 μl of RNase free water (the same 30 μl were put through the column twice). RNA concentrations were assessed before and after TURBO DNA-free treatment (Ambion by Life Technologies) with a nanospectrophotometer (Nanodrop, Thermo Fisher Scientific). An Agilent 2100 Bioanalyzer (Applied Biosystems, Carlsbad, CA) was used to further assess sample quality. RNA samples were stored at– 80°C until shipment on dry ice for sequencing.

### cDNA library preparation and Illumina sequencing

RNA samples were sent to Hudson Alpha (Huntsville, AL) for library construction, and Illumina sequencing. Sample quality was assessed using a Qubit 2.0 Fluorometer (Invitrogen, Carlsbad, California) on a 3X dilution (range for the reported concentrations was 5.64–16 ng/μl) and an Agilent 2100 Bioanalyzer (Applied Biosystems, Carlsbad, CA) on an 11X dilution. Samples were prepared according to standard Illumina paired-end library protocols prior to sequencing. Briefly, samples were normalized for Poly A RNA-Seq library reactions and stored in a 96-well reaction plate at -80°C. Up to 500 ng per sample (largest amount under 500 ng from a maximum 50 μl per sample) was used for the Poly A selection assay. Oligo-dT selection was followed by fragmentation, random priming, and cDNA first and second strand synthesis. Sequencing occurred on the Illumina Hiseq 2500 platform running v4 chemistry to generate ~25M paired end reads of 100bp in length for each sample.

### Data processing and transcriptome assembly

Prior to assembly, raw fastq read files from Illumina sequencing were assessed with FASTQC (v0.11.2) software (Braham Bioinfomatics) to determine read quality. Quality processing of reads was performed with Rcorrector [116] and the python script FilterUncorrectablePEfasta.py from Harvard Informatics Transcriptome Assembly Tools (https://github.com/harvardinformatics/TranscriptomeAssemblyTools) to identify and remove erroneous k-mers and. Cut-adapt [117] and FASTQC were wrapped with Trim_Galore (https://www.bioinformatics.babraham.ac.uk/projects/trim_galore/) to remove adaptors and low quality bases. The trimmed read files were mapped using Bowtie2 against the SILVA database [118]and reads that matched rRNA sequences were removed. Remaining overrepresented sequences were removed with the RemoveFastqcOverrepSequenceReads.py script from Harvard Informatics Transcriptome Assembly Tools. These processed reads were assembled into a *de novo* transcriptome using Trinity v2.2.0software [119] on the Bowdoin College High Performance Cluster (Microway Quadputer system containing four Intel Xeon E5-4620v2 2.6 GHz eight core CPUs for a total of 32 CPUs and 256Gb of DDR3 1600MHz ECC/Registered memory). For the assembly, reads were pooled from all prothoracic ganglion libraries and the minimum sequence length was set to 200. Trinity was run with the following parameters: library normalization with maximum read coverage 50, and RF strand specific read orientation, maximum memory, 250GB, and 32 CPUs. Raw reads were mapped against the *de novo* transcriptome using Bowtie2 software[120].

### Interneuron backfills

In order to control for the results of future single cell RNA-Seq experiments, in which individual AN cells will be fluorescently labeled and collected, prothoracic ganglia were backfilled using conjugated biocytin Alexa-fluor 488 (green) from Invitrogen. Backfills of AN axons via the neck connectives were performed on control crickets 24 hours post-control-amputation as described previously [13]. Crickets were stored at 4°C in a moist chamber for 18–24 hours to allow the backfill to complete. Backfilled ganglia were then removed and processed as described above.

### *In silico* transcriptome mining and deduced protein vetting

BLAST searches for *G*. *bimaculatus* transcripts encoding putative axon guidance proteins were conducted using a protocol that has proven highly effective for the discovery of sequences encoding a wide variety of arthropod proteins [121–123]. Specifically, tblastn searches of the *G*. *bimaculatus* transcriptome were conducted using known *Drosophila melanogaster* axon guidance proteins as query sequences. The complete list of proteins searched for in this study, as well as the specific queries used for each search, is provided in Table 4.

A workflow developed to vet the identification of a variety of proteins was used to characterize the sequences deduced from the *G*. *bimaculatus* transcripts [121–123]. First, nucleotide sequences were translated using the “Translate” tool ExPASy (http://web.expasy.org/translate/)) or the program Geneious, version 10.2 [124], and assessed for completeness. Proteins listed as “full-length” exhibit a functional signal sequence (including a “start” methionine) and are flanked on their C-terminus end by a stop codon. Proteins described here as “partial” lacked a start methionine (referred to as C-terminal partial proteins), a stop codon (referred to as N-terminal partial proteins), or both of these features (referred to as internal fragment proteins). Next, to confirm that each of the *G*. *bimaculatus* proteins identified here is most similar to the *D*. *melanogaster* sequence used to identify the transcript encoding it, each was used as the input query in a BLAST search of annotated *Drosophila* protein dataset present in FlyBase (version FB2016_05; [125]). The online program MAFFT version 7 (http://mafft.cbrc.jp/alignment/software/; [126]) and Geneious were used for all protein alignments. Protein domain number, identity, and relative position were assessed by running a conserved domain search in NCBI [127] on August 14^th^, 2017. Concise results were viewed, and specific hits, but not superfamily information, were used to create representations of proteins included in the Figs.

### Sequence confirmation

Additional prothoracic ganglia were removed from adult crickets, pooled, and RNA was purified as described above. cDNA was synthesized using reverse transcriptase (Thermo Fisher First Strand Synthesis kit) and used as template for PCR reactions. Geneious was used to design primers against select the top Trinity hit for *slit*, *robo1*, and *PlexB*. *Sema1* and *2a* have been previously sequenced and uploaded to NCBI and were used to confirm sequences predicted from the RNA-Seq. APCR products were sequenced using dideoxy Sanger sequencing (MDIBL). Geneious was used to align sequences and extract consensus sequences. ORFs are presented, and predicted bases that were not confirmed by sequencing are represented by “?s”.

## Supporting information

S1 Fig Predicted protein sequences for the top Trinity hit for each protein candidate. (DOCX)

S2 Fig Conventional sanger sequencing for select *G*. *bimaculatus* candidates. (DOCX)
