# Supplementary material for: De novo assembly of a transcriptome for the cricket Gryllus bimaculatus prothoracic ganglion: An invertebrate model for investigating adult central nervous system compensatory plasticity
Source: PLoS One. 2018 Jul 11;13(7):e0199070. doi: 10.1371/journal.pone.0199070 (PMC6040699; doi:10.1371/journal.pone.0199070)
Supplement: S1 Fig — (DOCX) [file pone.0199070.s001.docx]

Supplemental Figure 1.

A) Putative *Gryllus bimaculatus* Slit (TRINITY_DN152282_c0_g1_i2)

MMADRRTKVLSFLIFTLVLVKLCDSSPNNDVLTAASSSSRTGVPEELIHCPWACSCSGLTVDCSHRGLTQVPRNLPTDAERVDLQANNLTVLFETDFEEMTNLRILQLMENEIHTIERGAFQDLLALERLRINNNRLRHLPDGLFGNMPNLLRLDLSHNQLEIVGRKTLRGVTTLKNLQLDNNQITCIDELALRGLSNLEILTLNNNNLTSLAKDIFEDMYWLRTLRLSDNNLICDCHLAWLARWLRRYPRLALYTRCFSPHHLKGQNVADLHDQEFKCSGLVERPTGECLSEPQCPHPCRCADGIVDCQEKALTKVPDHLPEGTTELRLEQNQVTDISAKAFFPYKRMSRIDLSKNQITKIAADAFQGLTSLTSLVLYGNKIKDLPAGVFYGLTSLQLLLLNANEISCIRRDTFKDLHSLNLLSLYDNNIQSLANGTFDFLKSIQTLHLARNPFICDCNLRWLAEYLHRNPIETSGARCDTPKRMQRRRIEALKDEKFKCVEEYRTRLAGECLIDNACPNGCSCEGTIVDCSGRSLKEIPKDIPMYTTELLLSDNDLGRLKSDGLFGRLPNLQKLDLRRSKITGIEENTFEGCSRVSELLLSENKIHEVHNKMFAGLGNLKTLSLYDNQITCVMPGSFDALTSLHSLNLLYNPFNCNCHLAWFAEWLRKRNLSSGSPRCNSPPRVKDVQIYELPHHEFKCTSESEQGCLGDNYCPPKCTCTGTVVRCSRVRLKEIPRGIPSETSELYLDVNEIPVIHGNRLSHLKSLTRLDLSNNQIQVLSNFTFANLSKLSTLIISYNKLQCIERDALAGLTSLRIISLHGNDISMVPEGAFADLQSITHLAVGANPFYCDCSLKWLADWVKRDYVEPGTARCAEPHNMRDKLLLTTPASAFVCKGHISYEILSKCNACFSFPCANSGTCEALPQRKYVCRCAPGFHGQSCQHMIDACFGNPCRNGGTCKVMEEGRFSCHCPPGYTGDRCETDINDCLNNKCENNATCVDLIQSYECHCQPGFMGEYCETKIPFCTLEYNPCQNGAQCVNIITDYKCECLPGYSGENCSVNVDDCVNNICQNGATCIDGVNDYVCKCPGDLTGKYCEIAPMVAMLYPQTSPCQHHDCKNGICFQPMGSNDYVCKCAPGYSGKRCEYLTSLSFVHNNSFVELEPLRTKPEANVTILFATEQENGVLLYDGQNEHIAVELFNGRIRVSYDVGNYPVSTMYSFEMVSDGKYHVAELIAIKKNFTLRVDHGQARSIINEGKHDYLRLTSPLYVGGIPSEPGQEAFTQWHLRNLTSFHGCMKELWINHKPVDFVNAARQQKVTPGCSLMQDEEEQMEEDRAVIEDDDVEMNACHNNQCRRGSKCIPKRQGEYACRCRPGWGGRYCEQAPSCRKEQTREYYMENGCRSRKPVKLAKCEGNCGSSCCRARKTKRRKVRLICNDGTRYTTDVDIVRKCSCTKKCY

B) Putative *Gryllus bimaculatus* Robo1 (TRINITY_DN172482_c0_g4_i2)

MALARKIAASTAETLALLALLLTAVPAPVFAQYRTPRITEHPSDIIVAKNEPVTLNCKAEGKPEPSIEWFKDGEPVRTSPSDNKSHRVLLPAGSLFFLRVVHGKKEQDGGVYWCVARNTAGQAVSRNATLQVAVLRDEFRPVPKDTRVAAGETALLECGPPKGHPEPSLEWRKNGQVIEMENSKRLRVVDGGNLMITDVRQGDEGKYQCVAQNMVGVRESNPATLTVHVKPFFSKEPSDVTVLEDSAVRFECRVGGDPAPNILWRRDDGKMPIGRAQILDDKSLHIEHVTPDDEGLYICDAENVVGSVSARASLTVHSPPTFITKPQDQKVGLNGIAVFECVAKGNPPPSVFWTKEGSQVLMFPGNSYGHLHVTPEGSLRIQGVQREDAGFLVCSALSVAGSNTARAFLQVTSVDDVPPPIVQIGPVNQTLPLKSVATLPCQATGTPPPKIKWYKNGSPLAGQGPRITVLETGTLHIDDLQLTDSGLYTCTASSESGETSWSASLTVEKNPSPSGPGLHRAPDPSLFPASPPQPRILNVTESSITLSWRQGSSSATGTAGTTEPPIGYTVEYFSSDLQTGWVVAANRVATDTITIGDLKPDTSYVFLVRAENPYGLSIPSPLSESARTQRADRRAVPRYELDEARARLSAKVVTLRDVQPLNSTAVRLLWDILSGEGYVEGLYVRFRDLSGGSQKYNMVTVLNAGATSYAVTNLRKYTKYEFFLVPFYKSVEGQPSNTKNVQTLEDVPSAPPDNIQVGMINTTAAYVKWSSPPPAHHNGVLLGYKIQVKGNGTKVLAQMTLNASTTSVLLNNLTTGGAYTARVAAYTRRGLGPFSSPVSLIMDPALLHQYPPRAHPSEGSGGSVVQETWFLVLMASLVLALVLGFVGMLYLRRRQAMGKELGHLNVPVVNANDISQLNLMNGKETLWIDRGWRPADKDAGLPSETKLLNTGAPSLELVSNTTDYAEVDTRNLTTFYSCNRNNKDNVPEIPAPYATTTLINSIPRREMDNGHMFMPITVGGPGEAKTSSSSDSCVKPDLSSLDTNPEPGNKSSSPSSEVGNMYADDGNMQLRRLPLHQPPSQVRKFPVGGQQVLPNWSELLPPPPEHPPPSTMNEGISNNRLQMPSGSSSNRGVSPNFHPHTGNFNPNSPLLGKRNTCSREGTPLGHLSHPGEGNHAVPMGNTPPLPPVRGGSSCSSAGYTGPWVPNNPAEQNMYGSNSGGRYSLMPPQQHPPPVPNFPMGFGGAGGSSTGGSSNHSGSQHPGHHHNHHHHHHHHHAKSPSGNPNGNPHLEESIYESGSLVYGDTGGGHDDYQQHNSSTGGTSQIGSSAGYGAMDRGIQSSLPSLASENLSSRLHPSVAAQMAMDLGNPSDGEAVMGDYSDCDRWRSPGGEDSTTAGSWDEDRGSCSSGDASDTCCSCSESSCLYAETTELANQNPAGLPPQVGGPCTHNAAANARRHVRRQYPPHRTSASGSGRPVSPSYSTDSNYSCARPPPARSHLRPAVATSTASSVGDSSPYTSQTATPQHCRRDDTPAYAKPNYPTSQTSRNNTLGSGGTLVGSSAGSQRLKNLGNVFQNSSFPNTGTSTTSSSSPGEGNQPSEVVR

C) Putative *Gryllus bimaculatus* Robo2/3 (TRINITY_DN174950_c3_g2_i1)

PATLNCKAEGSPPPVVEWFKDGEPVRTSPADAKSHRVLLPAGSLFFLRVAHGRKESDGGVYWCVARNGAGEARSRNATLDVAVLRDDFRAEPKNTRVAQAETALLECGPPRGHPEPVVFWRKNGQALDLDQSKRIRLVDGGNLAIQDARQTDDGRYQCVARNIVGTRESSIALLKVHVKPYLIRGPSDAVALAGGSVEFQCRVGGDPPPDVLWRRTAGGGGMPLGRVHILDDRSLRLENVEPEDEGEYSCEADNDVGTVTASATLTVHSPPSMTVRPVDVSVEVQRDAVFECAMVGNPRPSIFWSVEGNRSLLFPGARIDRFAASATPEGRAVLTLQAVTRNDSGTVVVCTAVNAAGSAVWRARLTVTSPEDRPPPVITLGPANQTLPVKSMAVLPCRATGVPTPVITWYKDGAAVLASPRVNVSDSGLLQINDLEKRDGGLYTCVASSRSGKATWTAALRLEQPTNPNIHFFRAPEPSTFPGAPSRPQVVNKTDSSVTISWTRNNKIGSSSLLGYQVELFGREQNAAGGSASSASNGRGAHIYPLGSGGGWVVSARRVQGPTYTQHHLMPGVSYTFMVRAENSHGLSPPSPLSEPVSVAPTGSAWPGHGEEQFLLNEARASLLAGHVVELTDAQPISSTAVKLVWEILNSEYVEGFYIYSRPLDGSRTPDSVNILTVLHAGGASGFQVKGLAKYTRYEFFLIPFFKTVDGRPSNSRTVRTNEDAPSEPPMHMEALLLNASSVFLKWKPPPPNTHNGILRSYQVIVRGNSVTNSNSVNGPEAKGNTSRILTNITVGASTPSLLLTNLTTGVTYIVQALASTRAGLGPPSAPFTLRLDHASRLLFKDQHHRQPVGHDPSLNPGMSGSDFLTETWFIALLGSMVAVMVLLFAAMLLVRRRQLLSKKTTLPNLHDSRSNGGVLATPLSLKAAVGLPHPLANASHVSLPHDSSLWIENRPGPPTSWRHSETSDKETCSMSESRLLNNGSVVTTTLNDYAETGMLKSAASPNSETTPAYAEVDATHAALTTFRGCDERCSGGQISSDGSGSPAPYATTTLIGSSRQHMNNLGWVQICPPVNDSDDPSYPNPACYFGRNVYSDTYFFSGHCNNGLNGNPGNMIGPSGSHNSCPGTGGPTRKAFSDVNHESLAGMPPPMNPPIPPAASASVPHTPSNTLRRGHRNLQFIRPQIQRQGGGVNGENSHMSSTNPQPPAFSNNSCPSSRDCQRFSDPPPDVVTSPQQPPPPINCRVSPQGPQSWKGQNGSSGVKPMLLLTSIPVGSDASQTSPSAQDLNHSTNSYNAHSLSSFVVPYPTYSPAARHGQYQPVYHQSSRSEPGSSLGNTGN

D) Putative *Gryllus bimaculatus* Comm (TRINITY_DN156272_c0_g2_i3)

MALAGPPSSPSSSPSHNFTVLVAEVADDGYDTLVADIWVGVVLTLMVLSCVCCVCSCLLYHKFQQWKRSVLAARGPPGEATEAPYADVESLPSYTIVSGLPSYEEALRQLRLARERRESLKSASDLAAPPPASPTAPAAPASPLA

E) Putative *Gryllus bimaculatus* Netrin-A (TRINITY_DN160784_c2_g1_i3)

GLPAMHYAVSDLAVGGRCKCNGHAARCVAGANGQLQCDCRHNTAGRDCERCRPFHFDRPWGRATARDARECKACNCNQHARRCRFNMELFKLSGRASGGVCLKCRHFTAGRHCHYCKEGYYRDPTKPIAHRKACKACDCHPIGASGRTCNQTSGQCPCKDGVTGVTCNRCAKGYQQSRSHIAPCVKIPKVPNVMQATASDSGEDGPPGSTDREQCGKCSAGTRRLNLIKFCRRDYAVLARVVAREAAGDWVRFSATVQQVFKRARESRLRRGALDLWVHAHDLACKCPKVKATKTYLVLGREAEGGRPGLTLSPRSIVIEWRDEWRRRMRRFQQRARDCP

F) Putative *Gryllus bimaculatus* Frazzled (TRINITY_DN172587_c0_g1_i7)

MVVLPSGALEISQVWPLDQGSYQCNASSLQQSQLSAPAVLSVDQDRELDPRPIPPRFIATPHASVVATEGHNVTLDCAANGPPEPDISWLKDGSSIDLKDRDSRFMKVGTGSLRISHVQEKDRGTYQCRAGNRADLVDATTTLEVEVPPRFVKRPKDREAFEKEDLELECSVYGKPEPKVYWMKNGDLIKPSDYMHVVNGNNLKILGLMRLDTGIFQCLAMNPAGNIQASARLQIYPPASELSGNANNPSSVLNPGSNLKHNRGPFFLDENDDDEDIDDDGDEDNDDDDDYDYNYEDNDDYDARLDEDSLDRFSKLKNPTPLAPPTDDAVAPVRDVPSAPEGLTAVIVATRFVTLSWKPPANPRGEILVYSVYYRKEGSPRERILNTTRSQLEEANIPGLMPDKLYHFRVVAYNEQGAGASSETLSIRTDPEVHVLGPPRSLAAIPVSPNALLVIWQPPVDAPAGPPQGYKLFYMEVGSAEEHRVETQETHFNLTGLTPYTEYSVWVIAYNQNGQGSSTEEVTARTLSAPPSDCPQNVTLEPTSSSSILVRWEPPPKEGRNGLITGYKLRFRKRDRRGRGGDGANTISTPGDRRSLALPELERGAHYQVRLWAMNVNGTGPPTEWFSVETFENDLIETTVPDSPHGLKTRAMVDSITVSWSPPRNQNIMVRGYTLGWGKGIPDVYTKLLDGKQRYYVIENLEPTSEYVISLRAYNEMGDGPPAYSSVRTLDNIAPEPITPLHPPVGLMAIVLSSRSVVLYWTDTTLSKNQQVTDSRYYVVRYTSQHHASSPRYKYFNASDLNCMIDDLKPNTQYEFTVKTVRGRRESPWSMVVLNMTQEDQPYSPPRDLTVVPVAENPTMVNMNWQPPKQPNGPITGYLILYTTDSSLPDREWVVEGIVGDRMTTTVKSLTPETTYYFKIQARNSKGYGPFSSVVSFTTGPSRNFYDADTRGAQMRDGRGIPQTIVYIVIGCSVVVFTVLVGLGILVCCKRRSAERSKKGYMKGAIKGKPGKQNIKPPDLWIHHDQMELKALQQQAGVSGSIGGPSGDGGGPSGVPATLPRTSQTMGSSDFDQDHHDGTKFHSNSLDKRGYVSSYIGQPMDPGAGRPVYPRTQYSISRAHVTLDPAAGATVAAVAPVSNTVPPEGPYALQGGYDAVGVGGANTHAVPLSMPQSSHQQHAAQIVPPSAYGAGMQAVGVAGEAGASGTLGKRTQGHPLKSFSVPAPPPQSAPSTPQQKHIAVSSVTIRPQGSCSPYKKAGHLASGTSGTSTPPPVTIPSAPGSAGSNKSGRPTPDSHAQMQPSYSTEELNQEMANLEGLMKDLNAITASEFEC

G) Putative *Gryllus bimaculatus* Unc-5 (TRINITY_DN149614_c0_g1_i1)

GEGAGEGGGAAGSPSLQQDFVDPQTGVRNVEAAINITRNDVEEYFGKDDFKCECVAWSSRGQIRSQPATVDVAYLKKQFDAPPYAQQVQLEQQAEVRCHPPPGVPPPRVYWLRNGVPLEPDTNLIVSSEGHLLVGQARLQDTANYSCVAENVAARRVSEPAQLTVYVNGGWSPWSPWSECSARCGRGSQKRSRQCSNPAPLNGGALCPGPSVQRTDCTTICPPVDGRWSQWSAWSPCGPDCRHHRRRACSSPAP

H) Putative *Gryllus bimaculatus* Dscam (TRINITY_DN173597_c2_g22_i1)

VPPRWIVEPTDKAFAQGSDARVECKADGFPRPVVTWKRAAGDTPGDYKDLKPNNPNIKVEEGTLTIANIQKTNEGYYLCEAVNGIGSGLSAVIFISVQAPPHFEIKLRNQTARRGEPAVLQCEARGEKPIGILWNMNNKRLDPKSDNRYTIREEILPNGVLSDLSIKRTERGDSALFTCVATNAFGSDDTSINMIVQEVPEIPYGLKVLDKSGRSVQLQWAAPYDGNSPIKRYLIEYKISKGTWEEHIDRVLVPGQQTVAGVFNLKPATTYHLRIVAENEMGASDPSDTVTIITAEEAPGGPPTGVKVTAEDQSTLQVTWKPPERDQWNGEILGYYVGYKLASSESPYLFETVEFSKEEGKEHHLKIDQLKTYTQYSVVVQAFNKVGAGPMSEDTKQYTAEGVPEQPPQDTTCTALTSQTIRVSWVSPPLTSANGVIKGYKVMYGPTDTWYDENTKDTKITSSSETILHGLEKYTNYSIEVLAFTSGGDGVRSAPIHCQTEQDVPEAPSAVKALVMSGDSILVSWKPPEQPNGIVTQYTVYVHEDSEEAKDDEPKSQKVPAFQMSYEASGLKENKRFEFWVTASTVIGEGQNSKSVTISPSTRVPAKIASFDDSFTATYKEDVKLPCLAVGFPAPEIQWKVKGVAFTADDRIRLLPEGSLFIKSVTRQDAGEYTCSVENTFGHDTITHQLVINAPPHAPQVSLSAMTTTTITMKLKAHPADTAPIHGYTVHYKPEFGDWETAQVPAQATKHTLENLWCGSRYQLYVTAYNGIGTGDPSDILNTRTKGSKPVIPDVNKFIEVSTNSITLHLSAWSDGGCPMLYFVVENKKELQTEWNQVSNNVKPGGNFVVLDLDPANWYNLRVTAHNNAGFAVAEYKFATLTVTGGTIPPLTVGDGSSNPLLPWLPSWVELNVIVPVGATIIVIVVGVVVICVALSRRARGPEQTRLRDDVVYNQSAGGASTLDKRRPDLRDELGYIAPPNRKLPPVPGSNYNTCDRIKRGHTGSFRSHHATWDPRHARHMYEELSHHPPGRRVPAHPMGSDETLYHRVGGMEDEICPYATFHLLGFREEMDPSKAVQFQTFPHQNGHERSHSGTMGPSSGNGHVHQRSGSQSMPRTNGRYSRVASQNGPNSNFSPGPEYDDPANCAPEEDQYGSQYGQYGAPYDHYGSRGSVSRRSVGSARNLPISGSPEPPPPPPRNHDPNNSFNDSKESNEISEAECDRDQLVSRNYGGESSTAQPQSHEDPAQVAKY

I) Putative *Gryllus bimaculatus* Ephrin (TRINITY_DN159904_c0_g1_i6)

PRRGGARGSALARMAPAALSGARLTSFLTTVLLVCLETVLLCSVSCTKSFHVNWNTTNPIFRIDNTDHIIDVNKGNIPFEYDQVNIICPVYMPGTHEEDAEKYIIYNVSKEEYDTCRITNPNPRIIAICDKPYKLMYFTITFRSFTPQPGGLEFQPGQDYYFISTSSKDDLHRRIGGRCSTHNMKVVFKVCCQPNDLQNQTSAQRPSSTTVITSVGTSPSVSAAVSTAATPTSTRPTSLKELDMPNYNVKSTTKKTNEYDNKHPNEVLKNEELTYNSAQTLAAGPVLWQLLTGTCATVLLAVRLVGR

J) Putative *Gryllus bimaculatus* Eph (TRINITY_DN159184_c0_g1_i3)

MGDQLRTLLLLLLASAALWSRVSAEQVVLLDTTQEPTLEWTRYPYGPQANTPGWVEESFTNFEKGINWRSYVVCDVAYNNVNNWLWTPFVERRTANRIYIEIKFTIRDCSLFPGNALSCKETFSLLYYEFDAATREPPPWEPESYRLIGRIAAGEGRFNTNSEVTINTEIKSIPVTKKGVYFAFRDQGACISLLAIKVYYITCPEVTVNFAHFPTTPTGREVTFIEQATGKCVDNAEEVEPPTYLCKGDGKWYLPSGGCKCKAGYEADEDKQTCNVCPAGTFKHNTGDERCQPCPNHSKALESGFAECRCNAQYYRAPKDPKNMPCTQPPSAPRNLTVNFVDQSTVMLSWQPPHFLGGRNDTVYRVKCNTSASGVIYSPKTVVFNSTEVRISGLNPVTTYQCQVFAENGVSELSGQSQSVDVTVTTEASVPSTITKVNITNVKSTEITLEWDAPVIGDDPEVESEVVEVYEVRGFPTSNSSNPIIARTQKQKYTFTGLRQNTDYSFQVRAKTQHGWGEYSKVVSKKTGQVLEHFGNTDMQVRLIAGATVGVVVFLVIVIIIAVVFLRSRGSDECNKKQPSDCDTLEYRNGEVTTPLFTAVGAASRTYIDPHTYEDPNQAVREFAREIDASCITIEAIIGGGEFGDVCRGKLKLSPDGRQEIDVAIKTLKPGSSDKARNDFLTEASIMGQFEHPNVIFLQGVVTKSNPVMIITEYMENGSLDTFLRANDGKFQVIQLVGMLRGIASGMQYLSEMNYVHRDLAARNVLVNAQLVCKIADFGLSREIESTTEGAYTTRGGKIPVRWTAPEAIAFRKFTSASDVWSFGIVCWEVMSYGERPYWNWSNQDVIKSIEKGYRLPAPMDCPEAIYQLMLDCWQKERTHRPTFASIVKTLDKLIRCPDTLRKIAQNRTANPLAPDAPDMTQFTSVEEWLNSIKMARYLENFERAGITSMDAVVRVTVKELTALGITLVGHQKKIMNSVQAMRAQISANLSEGFLV

K) Putative *Gryllus bimaculatus* Sema 1a (TRINITY_DN169697_c2_g1_i3)

AGVPPGARGRGLLALLLVTMGAAATVDAATGAWQENVRPKMYVQLGTNDVFRFTGNDSHTDFFRLVIRDGNSLLVGGRNLVHNLSLPELVENQRLVWYSPDEDVKMCVMKGKDEENCQNYIRILARTGAGRYLVCGTNSFKPVCRDYSIQSSGYVVEREKNGQALCPYDPAQNSTAVYVDSDLYTGTVADFSGMDPIIYREPLQTEQYDSMSLNAPNFVSSMTQGDFVYFFFRETAVEYINCGKAVYSRVARVCKYDRGGPHRFRNRWTSFLKSRLNCSVHGDFPFYFNEIQSTTELIEGSYGETTAQLVYGVFTTPPNSISGSAVCAFALQDITDTFEGNFKEQAQLNSNWLPVQSVKVPDPRPGQCVNDSRTLPDLTLNFIKTHSLMDESVPSFFGQPIVIRTSFHYRFTQIAVDPQIKTPGGKPYDVLFIGTDNGKVIKAVNADSADSNTEVSPVVIEEIQVFPPHVAVRNLKVVRDSSFDDGRLIVVSDSEVQSLRLHRCYSDKILSCSECVALQDPYCAWDKQSQKCRSVGAPRWNDEKYFYQSISKGVHSACPASKVMGKDAGSVGGLSSNYPKSFNHDSGRSSKDIQGGEVINIMHDEEEHTGPEVSAADSPMPQYSVETLAMAVVAGSVAALVVGFVTGYLCGRKCHKEEEDNLPYPDTEYEYFEQRQTVNSRLQPEPKLLPQEEVTYAEPVLVPAPGPNKLNSPKSTLRKAHNANHAAETLFQFSDNYTPPPRDPYAHQRGRDNFGTLRSQQGDGYRGGGGGGGGGGGRAPPG

L) Putative *Gryllus bimaculatus* Sema2a (TRINITY_DN173773_c2_g2_i6)

MARGRSALLRFALVACVLAELGVFTAVRAGYDNHWAFYYEQPCCGGHHLRHHKDHVREFSCGKMYYRTFYLDERRDSLYVGAMDRVYRLNLSNISHSNCERDSMSLEPSDVANCVSKGKSEHFDCRNHIRVIQPMGDGGRLYICGTNAHSPKDWVIYSNLTHLMRKEFVPGVGLGIAKCPYDPADNSTAVWVERGNPGELPGLYSGTNAEFTKADAVIFRTDLHNLTTGRSEYTFKRTLKYDSKWLDKPNFVGSYDIGQYVLFFFRETAVEYINCGKSVYSRVARVCKKDTGGKNILSQNWATYLKARLNCSIPGEFPFYFNEIQSVYKVPGDDTRFYGVFTTSMTGLVGSAICVFTLRDIQEAFRGKFKEQATSASAWLPVLSSRVPEPRPGDCVNDTETLPDTVLNFIRSHPLMDAAVAHEHGKPVFFKRDLLFTDLVVDRLKIDLIASIKEYIVYYAGTNNGRVYKVVQWYDEDAEDSRSRLLDMFDVTPGEPIRLMEISREHRSLYVASDHRIRQIDLVMCNRRYDNCLRCVHDPYCGWDKDSSSCKPYAPGLLQDVANRTVSICDSSVVKKKMVVTWGQSIHLGCFLRMPEVLSRQTVTWYHYSKEKGRYQIQYRADKYIETSERGLVIIAVTEADSGRYDCWLGGALLCSYNITVDAHRCAPPGKSHDYQKIYSDWCHEFEKYKMAMKTWERKQAQCSTRQNDSNQNAHPNEIYHRSPLV

M) Putative *Gryllus bimaculatus* Sema5 (TRINITY_DN165105_c1_g2_i8)

MVYCDQIDSTDFRTISQQDLLTSAHQFNEAGVTSYSQLLFDVSRNQVFVGARDALYRLSLWNLKLLEKAYWPAPSNKTSLCLEKGQTEEDCHNYIKVLLTNGKYLFSCGTGAFSPECSWREMENINKVQEWVKGVAKCPFSPKSNITSLLTMEGQYFVGSPMDFSGTDSAIYRSMTPVTLRTNQFNSKWLNDPDFVGSFEVDKFVYFIFRETAVEYINCGKIIYSRIARVCKNDTGGQLMLKENWTTFVKARLNCSLPGEYPFYFDEIQGMYYLEEEGLIYATFTTPSNSIPGSAICIFNMSAISLAFSGPFKHQDKPGSAWERHHIPHRSLLECQSSPHAHQLLDISLYQLMDSAVQPVTLSPLYTSELETLTHIAVDQVATKLHRSVHVLYVATSRGLIKKISVLPRTMETCVVEIWKPFEDSYPSPIKTLHYLKETNSVYVGADFTLLQITAHHCNRFKNMVACLNAMDPYCGWNNLKEMCTPAPDGDPLAKYWLQNSTQCPVLTHPVDGGWSSWSNWSPCTHLNDNEGSLSDHDKCLCSTRQCNNPAPQNGGVSCKGMSMRVTNCTVHGGWTSWSAWSACSQTCGTAVKTKRRTCGNPAPAHGGRVCVGQDRSEIYCTSNPPCPVLTPPPRDGQWGEWGEWNTCSQACGGGFRVRRRVCDSPPPENGGQDCQGCHLSYETCNMQPCPEGKKYSSWTPWLQVNNTPSGNGYVERRYRFMCRAPVDVATIRITQTKEEERICHSNANCLKIEGQSDPDERWSEWSSWSSCSADCGGGQQFRSRFCNGPEKCEGLSHMSRQCNTHKCNAEWSCWSEWSECSVSCGQGIRQRTRHCLMAGNHMHRGTNCEGPSVGQEHCEVTSCESLRGWESWTVWSLCDANGMQHRQRKCQFPNPDEHYCQGLDMETRMCIDDGREINDLAIPSSLDADAASTIPIPVVLGSCAAAFTAGTVLAALFCYFLFRQQRSRVPGSPHYISSKQNPYVTVPLKEVGSHARRTSSPVTSSTCSSSGGKSAGSGTPKLFTKPSEYETATIKRNSHSLANGHIRMDLDQDKFF

N) Putative *Gryllus bimaculatus* Plexin A (TRINITY_DN159247_c0_g1_i2)

MRGAAVLVLLGLSLGIVGSGSANIVKNFTDDGTERFNHLVVDKNTGRVFIGAVNRLYQLSPDLELVMRDITGPEEDSPECSVLDCPPAVVKKPTDNVNKALVIDYTTTRLISCGSVFQGICSVRNLHNISDVAQVVKEAVVANNSTASTVAFIAPGPPNPPVTQVMYVGVTYTGNSPYRSEVPAVSSRSLDRDKMFMIAETAVTTGTRMFVNSLARERYPIHYVYGFSSEGFSYFLTTQMRHTSPSPFISKLVRVCHDDENYYSYTEIPIDCIAESGKRYYNLVQAAYVGKPGSDLASDLGITAQDDMLFAVFSENDQSEGEVSSKPSDYSALCVYSLKSIRRKFMQNIKKCFSGNGSRGLDFISPSHTCVLTKLQTIGEDFCGLDVNTPLGGENPMAAVPVLEFGTHLTAVAATSTGDYTVVFLGTSTGHLKKVVVESATSALEYGDLVVDEGSPVNPDLHFDSQLMHLYVMTEKKVFKVKVQECSVYTTCWECLGAKDPYCGWCSLENKCSLRSDCQDAAKDPLYWISYKSGRCTTITTVTPNQLQRTTARTLELVIDNLPTLPGQFLCAFTALDKTLITNATRKSYGVNCTTPRTDLLPSIPPGQHHFTAKLSVRMTSGPDFVATNFTFFDCNTYSSCTQCVSSSFPCDWCVDGHRCTHDTAENCRNDILVTGISRVGPSYRSGPGFCPTINATGGSTEILVSSGIKKSIKVKVHIIGQFIVQTRFVCQFNIEGRVTSVNAQLLGDTIYCDSMEFSYTSRAPNITATFAVIWGGSKPLDNPDNIHVVIYRCRDMADNCGMCLALAEKYGCGWCQVSDRCEVKEQCDGAGVWLNRNQTCPNPEVTAFEPQLGPWEGGTNITIHGINLGKTYNDIYGGVSVAGIQCEPYDSLYIKTKQIVCRVDGPGTNEPRRGPVIVRVEDFRGESKHNYEFVDPVIESISPKFGPRSGGTRLRISGKYMNAGSRILAFIDDLPCEIIETKAEEALCITSASDRQRSGKLRMKFDKGDRVFDKELFEYVEDPTIESAESGVAGQIKIPKGIPAGGIKISVSGKNLAYIQNPQMYVYFKQKMFGSQCVVQSNSNMACASPEIEVKGPIDADDPLKLEYGFRMDNVSGVQNLSARTNTHFLLYPNPIYEPFDEEIKYYKSDYLTINGQHLDRACQESDVVVQIGNSFCNVTSLSRQQLTCRPPTVQPPAVDKDGNINPQELPEVVVVVGGRLRYKIGKLSYASPTAPNGLMSKPALIGVIAGIVLLVVVFVAFLIAYRRKSTESNRVLKNMQEQMDILELRVAAECKEAFAELQTEMTDLTGDLTSGGIPFLDYRTYAMKILFPNMDDHAVLQWERPELLRKDKGLRLFGQLIMNKTFLLLFIRTLESNRYFSMRDRVNVASLIMVTLQSKMEYCTDILKTLLAELIEKCMEGKSHPKLLLRRTESVAEKMLSAWFTFLLYKFLRECAGEPLFMLFRAMKQQVDKGPVDAITSEARYSLSEEKLIRQSIDFKPMTVYVSISQQAVFVSGLDPNTENVPVKVLDCDTISQVKEKALDTIYRATPYSQRPRKDDLDLEWRTGTSGRLILYDEDSTTKTEGEWKKRNTLNHYRVPDGASLNLVSKQSSIYNLSILSEKTDKSHKYETLNLSKFSSASPPLSRATSPLNHDHDGGLKSWHLVKHHDTDAQKEGERGNKMVSEIYLTRLLATKGTLQKFVDDLFETIFSTAHRGSALPLAIKYMFDFLDDQALQHGISDPEVVHTWKSNSLPLRFWVNLIKNPNFVFDIHKSNIVDSCLSVVAQTFMDSCSTSDHRLGKDSPSSKLLYAKDIPVYKEWVERYYSDIKMMSAISDQDMNAMLAEESRLHTSEFNTNCALHELYTYAVKYNEQLTVTLEEDEFSQKQRLAYKLEQVHNIMAAESNP

O) Putative *Gryllus bimaculatus* Plexin B (TRINITY_DN161683_c0_g1_i2)

MLRVEETVTTGPRDDSPLCHASGCDSDDIERSLMDNVNKVLVMDPTSATLIACGSVRQGACEKYNVHNISVQPEFIPVSIAANDEHASTYAFVGPEHYKPWAPPSNVLYVGTTFTNNGEYRHDVPAIASRSLSNLKLAEFSFSKQSLLRIDVKYRDHFLVKYVYGFNASEYAYFVIVQKQSPLPGQEEMGYVSRLARACISDSNYDSYTEVTLQCLVRDPSQADGVVSYNLVQDAKVTEAGVDLANSLGILRGDPVLVAAFSPPARSISSEPQRRSAVCVYSLHDIEAKFNENIHMCFNGSIKYRNMDYVSGLILDGKCPNAGSTGNILSFCDVGLKISGVVPISSHAALHLPNTSLTAVAAATTERHTVAFLGTSDGVIKKVLLRSASQADEYEQVTVDAGRAVLPDTTVSPRGDFLYVLSTGKISKMKVEHCSSFANCSSCLEAKDPYCGWCSLEKRCTVRSACQKATHSSPRWLSLGGGQQCIDFEQVLPDRIPISQMTTVQLTIRTLPELPAGANYKCVFGAAEPIDAAVTAFGLSCPTPPVAGRPAIPEARDHVLVPLSVRSSETNKDFVSRNFAYYDCGRHTTCMQCVRCQWACNWCVYENKCTHNTSTCQRTVVSGENNPAQLATHGASFCPRFHSPTGDILLPNNVPKEIVLEVDNLPHPQVGHTGFQCIVNIEGAKMMVPARVESNRFIVCDRTTYSYEANTGEYEASVTIVWNRNHHVDTINVTLYKCDILGSHREHPDCSLCVTRSAKYQCVWCGLTCSYSQSCQHIPVSECPKPRIDMIKPLSGPIEGGTLVTIEGSNLGLKEEDVKGKISIGSTPCELVHYEVSVRIVCRTGPSDGEIIAPVMVGNEAGYTESAVHFSYKDVQLLGVFPPMGPQSGGTQLAITGQYLNIGSSISAYLDHLPCHVNSTQASSSRITCITTRSSEPMRVQRLTLTIDGANRTLESNPFNYTLDPTIMEIKPLTSFVSGGRMITVHGTNLDTILKPEMVVYIDEEPTPINKTVCSVLNTAQMECPSPPVNRKFLLSARQRRSPNKPSRSMPKYGTDAQLQLRIGFIMDNVESVKDLEKHFQNLRSHLLYVDDPQFFRFPNMNKLYKGDTLVIEGVNLNLASDESDVNVTIGTQPCNVTSLASTQLVCSPPEVQPAGTDEIGVRTDTNLPLVVVRVGRSLRFSIGFLRYEVIKPYTFPPEAIAGIAAGTCFLVFLFVIVLYVYRRKSTQAEREYKRIQIQMDTLESNVRSECKQAFAELQTDMTDLTADLESSGIPTLDHKNYIMKVFFPGVSDHPILNDPKIRVNGPRTNYDAAMLQFEQLINNKYFILTFIETLEAQKSFNIRDKVNVASLLMVVLMGKMEYATDVLCSLLLRLIDKSVCTKHPQLMLRRTESVVEKMLTNWMALCMYNYLKDYAGSSLFLLFKAIKHQIEKGPVDAITHDARYSLSEERLLREQIEHGVVTLHVVQDDLDEKIQCKVLDCDTISQVKSKILDALYKNTPFSMRPSIHEVDLEWRHGRGGHLTLQDEDLTTKSTGGWKKLNTLAHYGVKESAVMSLIPRQNDSFNANCKQPCQNCTGTYFTNSLSPIITANGDVEAGSNLRIYHLVKPIDEHHYQNNKTSERTHKAIPEIFLTRLLSTKGTIQKFVDDFFNTILTANDALPPAVKWLFDLLDEAARRHSIADPEVVHAWKSNSLPLRFWVNFIKNPDFIFDINKTTTVDSCLSVIAQTFMDSCSTTEHRLGKDSPSNKLLFAKDIPHYREMVSHFYCDVQILPQITDQEMSTAMQQLSISQLGEFDTVSALKELYIYVTKYSDQILDALDVDPYCKKMHLAHKLENVACTLEGEETSTC

P) Putative *Gryllus bimaculatus* Off-track (TRINITY_DN160729_c0_g1_i15)

TDAGAVRVDDGRLLVEAASARDHAGAYTCVADNLAGSREIALTLVVATQPRIINPPTQDPPSGAVYEEAKAVFHCEYEAMESPISTVEWLKDGILISEDRHRFKIQRGKSNSTLVINAVTMSDEGVYACQVNTIGFAPVVSENASLSVKERLKFSPKPVSKKLELGTPSTVYCRAQGSHPPTIKWFKSCQDTKQCKTSVDLPSHVQDINGTLEFRNVTLEDRGNYTCMATNKQGSISATIDIDVIVTPKFRVPPQNVTAYEGYPVILHCAAEGLPQPTIKWDRNSNLSALPSGFEVLSNGSLWSREVHISDGGKYGCTAGNSGGFKREEVTLIVESNEGYQPSEGIEQGETMMTKTVGITLGAAAAYMVLVVGLMAWCRHRRRKRKQAGLDAPAAEVGKVENGDVIMTDHHETKEHRKHEKKEKKERPRSDGDTTQSQGSNHSKKSKTGLDRLNFPRSDLHSFVLLGRGQFGEIQLAQARGIKEGSDVVVLVKALQPTRDDIILQEFKREIDLFTKPSHPNVAKLIGLCRNAEPNLMV

Q) Putative *Gryllus bimaculatus* Guanylyl cyclase at 76C (TRINITY_DN166112_c0_g1_i2)

AAPTTPPLPSTTSAPPLANSTALVPSTLSPPLEPLVNLTVGYLPACRGALADRQCMLISGAMTLALEQINNRTDLVPWARLQLRWNDTKSDPVVATRAITEMVCEGVAAFFGPEGSCHVESIVSQAWNIPMIAYRCSDEKPSSVPTFARTEPPDTQVTKSILSLLKYYDWKKFAVIWEEPWEAVAMTLMRQAKELYNSTITHQRQLQDPAKCCISNEECCRNTFWYQIINESKNITRIYVFLGLPRSLVNMMKQMHDLRLFDKGEYMVITADMMPYTHRETLKFVWDTVHMNNITCTEDAVKRGRSLFIVGLTPPTSHNYQVFADTVEHYNALPPFNFTTPHSLKIFRKHITIYASYLYDSVLLYADALHTVLENKQREGVLTAQMIESLAKNGTLIIQTVKDRGQYTSITGAVIKIDHNGDSEGNFSVIALVPEPIQDANYTCDFHFRPIGTFSFTDNSKLEYNVNDGTPWWIGSTRPPDEPTCGFDNQHCPDDSRSSSMYAAATLAVLLFCALVVTLSIYRKWKIEQEIEGLLWKIDRLDLHGYFGNDIVASPSKMSLASAASCESRCGQQLFAPTGTYRSVIVRIKELKFSRRKDISREVMKEMRLLRDLRHDNINSFIGACIEPMRILLITDYCAKGSLYDIVENEDLKLDKMFISSLVHDLIKGMIYIHKSALGCHGNLKSSNCVVTSRWVLQITDFGLHELRHSADNSGSIGEHQHFRSLFWKAPEMLRDPSAHILACRQKADVQAVQKADVYAFAIILYEMFGKKGPYGGCPLEPKEIIEMVKKVPKPEQKPFRPDLSLLEMEETPCEDFVIDCIQDCWSEAPENRPDFSTLRTRLRPLKEGSGRHRNIMDQMMEIMEKYAENLEALVNERTRQLAEEKRKTVDLLNRMLPAPVADQLTKGYGVEPESFETVTIYFSDIVGFTAMSAESTPLQVVNFLNDLYTLFDRIIKGYDVYKVETIGDAYMVVSGLPIKNDDRHAGEIASMSLDLLDAVKNHRIVHRPNDILKLRIGIHTGPVVAGVVGLTMPRYCLFGDTVNTASRMESTGEPLRIHISPQCRTALDKLGGYLVEERGLVALKGKGEVRTFWLTGATDCAVQRREVALDEMPPPLFCRPRRSPRLCAESRQAS

R) Putative *Gryllus bimaculatus* Mical (TRINITY_DN175376_c0_g1_i11

MEPRKQPVSPEVALASDVFDQFCNASTLKTILGHHRHLCELLRIKPTAFPQFYPKLKLKLRSWKAQALWSKFDKRASHKCYNRGKACPNTRVLIIGAGPCGLRTAIEAQLLGAKVVVVEKRDRFSRNNVLHLWPFVIADLRALGAKKFFGKFCAGSIDHISIRQLQCILLKVALILGVEIHEGVGFEGLIPPPADQSSEKIGWRAETSPPDHPVSQYEFDVLIGADGKRNTLEGFKRKEFRGKLAIAITANFINRHSEAEARVEEISGVAFIFNQKFFKDLYSATGIDLENIVYYKDETHYFVMTAKKHSLIDKGVILQDYTDTAKLLGLENVDKNALQNYAREAADFSTNYMLPHLEFAVNHYGQPDVAMFDFTSMFAAENASRVVERHGYRLLQILVGDSLLEPFWPTGSGCARGFLSSMDACWAIKSWGGGQVTPLDVVAERESIYRMLGQTTPENLHRDYNSYTLDPHTRYPNLNIRAVIGLQVRNLYDTDDPGSIEQPLPALASSEAPKKRRRRVKDSQVHPDTLLLWLKKQVALYDVKVENMTDSFKNGLVLCAIIHRYRPDLIDFPSLSPDDIAKNNQLAFDTLERELGVPPVMTGQEMEQCDVPDKLTMLSYLSQIYDTFRGEIPHIKHPKLELDPEEEKPPSRVMQLRQLNTEKKVALLNRITNQHAQHHHAHVHTHRKRSSAERDALAQSTSPGERHHHDFLRRRPNKRRSMERDKDRDSRLVGDTKRGDKDEDFSGRVKSLEEKLQGSRPGTDKKPKDLYRAIGKIEKSDWNVQVLEKKIQENKMGSYKHHNRPESVPKWDKQQFDDKRQAVQDKIQRRNPAANNKSDRYAHIDVKLQELEKKLKEGNVRDAGPRGANKVSAMAEQFKLKNQDTDAPPIQKSNSRAALVLPAQGGSEICHFCNKRVYLMERLSAEGKFFHRGCFRCEYCGTGLRQNNYLFDREGRHGSRFYCVQHFGMPGKLRARRKSEELRTGPFKENIPTSQKSPSKTPEKAKSLCETLSKLDASRDAVDMRATPERIEFENLAGATTDVEEAPSEMDEDEWTDRNFGASAAEGSSDSVSDISDSDDDMPNEVFEEAIQQPLTADETLRLAESWQRRYSKTPSKDLQNGESCNAEGKKDIPAAATTTREHEADTGESKLTSKPAKNITKTANNDDEEDSESYEYDESDGELSYREYESDGDGSDTATEGEEQNDADAKARELRRQEVQLQVPQRPPRSRHDSDTNTGSETEVASDYSTDTDSEEEEEEEEEEEIENSATEISTDSEFEHDGTTPTQHNIPSILIDNPALFRRSLPEEPKKVQVRSGHINGTVKPQTPAIQNAKNARTDGVRLEISPLRPETESKSSSLTNLASGNNILNTKNNNNLASSKLAAPTPLINPHKGDYFLNRTQSTEGIASKLSLQLKKKYLLGASDVGGSVKKSGSSSTLDSKVKNFVDVISEHQKKLNPAPEPSPTMQAFLQGTSNLRVNTIPNNPLSPPSPTLLNAPLKLPPSPSQTSASPRTQKEIPQLNEGFEHICGLGKKDENAESLSSLSKKDEFEHVCSLGKKQENALPATSLSKKEEGFEHICGLGKTNEDTSAKKESSPETSCKIPETVKSESPVNGLSSDVHQENVLTTEQYVKLEESSDISSHEKLVDKTDAQKMNNVINDQESYNNLDCRPRSPAHETSIVVPDISWNTGHKTEDIETDSLSSCGSGSDVEEEGDSDDHRSRSPSPVLKREMTPPRVEIHNSSGELMEDEYGHEGDKPQENSNKDDHNIEEDTTVQRVFSDAVGSSEDVNLRNKKRVEPLVFENKRASLTSDRSSPSTPPSARGDESESFHHEGTLAALTETELSDWAQDGDAVVSEDLEDVEFNINPEFVTIRRHRKTKTNRNAIRGNSASAFAKIARGEDFGDFDEDPGHVCGKERSGSQTESNKAPSSILSNIDNIEFMDTGGEEEDEDCSDTGDTEELLDARNKVLLRNNGYVQFVDTEDDMATPVAESPRQIVPAPVLPTFIVSECEERSGEHCDSPEIFEAVNKELVTASAGEEEESSLQPEGTTTEETTTTSELATVKDNLNADDSDVFFTPKQELSNNEGRSPVSPNDDLRGQEYDEYVKRLQGRISPFHNVRDSIDVRKSRRKIGNGKVNEHIKPQECISEESTSDHGAGEKIVPSLSSPINIYNSPSTSRKLEEISRERSKQKDLIHEMVLAKHLSQRKSSQERRQRKSSKGLQTPPSSISSSQFSSNDTFQTPLSNSSEVPASPKESVITLETPSLALPVVETEVIDSNRSIHQDLEVPFVDDSVLVTEETEQFFTPLTSFKKEPHHKPRPVSVHSSFRAEQKPIKYVNRYSFLPDTPLTNPEAFSLPDIRKALFKTPDEPFSPPVAPPRSRHDRTAGQEQDREELGERTCMKSSEELGFSPDDYITTLKKKSRRPGLERSLSQPEEAATARARLHERVDHILQPDASSSMTGSSFQNMQTSLTSTASPTSSIASPLSTPHSKTTFYLDANHSSPTSGETSLNSTKEQKKSKDPERRKSIIQAVSDFFHKKKDASATPSPPKTPTTSGSAGGSSKDKFRFKFSRLKDKSRVRKSYYICNLYENIILVVVLNYILNIIKII

S) Putative *Gryllus bimaculatus* Protein kinase, cAMP-dependent, regulatory subunit type 2 (TRINITY_DN165220_c0_g2_i1)

MSRAEAGRITVPDELRDILLEFTISYLLEQPGDVVEYAVEFFTKLRESRATAMVRLDEEQAVSPDESVLSMEEEPPVGRFNVRRKSVFAEAYNPEEDDDDVEKIVYPKSDVQRQRLAESVKHIFLFRSLEKEQMNEVLDAMFERVVEPGDYIIKQGDDGDNFYIIESGIFKAYVADGPNQKHIHTYENSGSFGELALLYNMPRAATIIATTAGSLWAMDRTTFRRILLKSAFKKRKMYEQLIEKVPMLKTLQPYERMNLADALVPRRYSDGEVIIQQGDVADGMYFIEEGVIRITIVGDNGQEVEINRCTTGGYFGELALVTHKPRAATAYAVGDVKLAFLDVEAFERLLGPCMDIMKRNIDDYEDQLVRIFGSKSAVSDIR

T) Putative *Gryllus bimaculatus* Rac 1 (TRINITY_DN170604_c0_g3_i2)

MQAIKCVVVGDGAVGKTCLLISYTTNAFPGEYIPTVFDNYSANVMVDGKPINLGLWDTAGQEDYDRLRPLSYPQTDVFLICFSLVNPASFENVRAKWYPEVRHHCPSTPIILVGTKLDLREDKETIEKLKDKKLAPITYPQGLAMAKEIGAVKYLECSALTQKGLKTVFDEAIRAVLCPVPAPKPRRKCVLL

U) Putative *Gryllus bimaculatus* Rho 1 (TRINITY_DN165708_c0_g2_i4)

MAAIRKKLVIVGDGACGKTCLLIVFSKDQFPEVYVPTVFENYVADIEVDGKQVELALWDTAGQEDYDRLRPLSYPDTDVILMCFSIDSPDSLENIPEKWTPEVKHFCPNVPIILVGNKKDLRNDPNTIKELGKMKQEPVKPEEGRAMAEKINAFAYLECSAKSKEGVREVFETATRAALQVKKKKKGRCRLL
