# Supplementary material for: De novo assembly of a transcriptome for the cricket Gryllus bimaculatus prothoracic ganglion: An invertebrate model for investigating adult central nervous system compensatory plasticity
Source: PLoS One. 2018 Jul 11;13(7):e0199070. doi: 10.1371/journal.pone.0199070 (PMC6040699; doi:10.1371/journal.pone.0199070)
Supplement: S2 Fig — (DOCX) [file pone.0199070.s002.docx]

Supplemental Figure 2.

A) Dideoxy sequencing of *G. bimaculatus* *slit*

GAGACTGGAACAGAATCAGGTCACGGACATCTCTGCAAAAGCATTTTTTCCGTATAAGAGGATGAGTCGCATAGATCTCAGCAAGAATCAGATAACAAAAATTGCAGCTGATGCATTTCAAGGTCTAACATCTCTAACATCATTAGTGCTCTATGGAAACAAAATAAAGGACTTACCAGCTGGAGTTTTTTATGGATTAACATCTTTGCAGTTGCTATTACTGAACGCTAATGAGATATCCTGTATTCGAAGAGACACGTTTAAAGATCTACACAGCCTAAATTTATTGTCGCTGTATGACAACAATATTCAGTCATTGGCTAATGGTACTTTTGACTTTTTGAAAAGCATTCAAACACTGCATCTAGCACGTAATCCATTCATATGTGATTGTAATCTTCGCTGGCTGGCTGAATATTTACATCGAAATCCAATCGAAACTTCCGGTGCTCGATGTGATACGCCCAAGCGGATGCAGCGCCGGCGCATTGAAGCACTCAAAGATGAAAAATTCAAATGTGTAGAAGAGTATCGTACACGACTTGCCGGAGAATGCCTTATTGACAATGCTTGTCCCAATGGGTGTTCATGTGAAGGAACAATTGTTGACTGCTCTGGTCGCTCTTTGAAAGAAATACCTAAGGATATACCAATGTACACCACCGAGTTGCTACTAAGTGATAACGATCTTGGGCGT????????????????????????????????????CTTCAAAAATTGGATCTGAGGCGCAGCAAAATCACTGGTATAGAGGAAAATACTTTTGAAGGTTGTTCACGAGTTTCTGAATTGTTATTATCTGAAAACAAGATACATGAGGTACATAATAAAATGTTTGCTGGACTTGGAAATCTCAAAACACTATCTCTGTATGACAACCAGATAACCTGTGTGATGCCAGGATCATTTGACGCTCTTACATCACTTCATAGTCTCAATTTGCTGTATAACCCATTCAACTGCAATTGTCATTTGGCGTGGTTCGCTGAGTGGCTGCGCAAACGAAACCTGAGTTCCGGAAGCCCAAGATGTTATTCCCCTCCACGAGTGAAGGACGTTCAAATTTATGAGCTACCTCATCATGAATTTAAGTGCACTAGTGAATCTGAACAAGGATGTCTTGGTGATAATTACTGCCCTCCTAAATGCACATGCACTGGGACAGTAGTTCGTTGCAGCCGAGTTCGCCTAAAGGAAATTCCTCGCGGTATTCCTTCGGAAACATCAGAACTTTACTTGGACGTCAACGAAATACCAGTTATTCACGGAAATAGACTAAGCCATCTGAAGTCTCTGACAAGGCTAGACCTAAGCAACAATCAGATTCAAGTTTTATCGAATTTTACTTTTGCAAATCTCTCCAAGCTCTCTACTTTGATAATCAGCTACAACAAATTGCAGTGTATTGAGAGGGATGCTCTCGCTGGCTTGACGTCTTTAAGAATAATATCACTACATGGGAATGACATTTCAATGGTACCAGAAGGAGCATTTGCTGATCTGCAGTCTATTACTCACTTAGCTGTAGGTGCAAACCCATTCTACTGTGATTGTTCCCTGAAATGGCTGGCTGATTGGGTGAAGAGAGACTATGTAGAACCTGGAACGGCACGATGTGCAGAACCACATAACATGCGAGATAAATTGCTACTAACTACACCTGCGTCAGCTTTTGTATGTAAAGGACATATCAGTTACGAAATTCTCTCCAAATGCAATGCATGCTTTTCATTTCCATGTGCCAATAGTGGCACTTGTGAAGCACTCCCGCAAAGAAAGTATGTTTGTCGATGTGCACCTGGCTTCCACGGGCAGAGTTGTCAACATATGATTGATGCGTGCTTCGGTAACCCGTGTCGAAATGGAGGAACATGTAAAGT??????????GGTCGCTTTAGTTGTCACTGTCCACCTGGCTATACTGGCGATCGCTGTGAAACCGACATAAATGACTGTCTTAATAACAAATGCGAAAACAATGCTACGTGTGTTGATCTAATACAATCCTATGAATGTCACTGCCAACCTGGTTTCATGGGCGAATACTGTGAGACCAAAATTCCATTTTGCACATTGGAATATAATCCTTGCCAAAATGGAGCTCAGTGTGTTAATATAATTACTGACTACAAATGTGAATGCCTTCCTGGATATTCAGGAGAAAACTGTTCAGTAAATGTAGATGATTGTGTCAACAACATATGCCAAAATGGCGCTACTTGCATTGACGGAGTTAATGACTATGTTTGTAAGTGCCCGGGAGACCTGACCGGCAAATACTGTGAAATAGCGCCAATGGTAGCAATGTTGTACCCACAGACATCCCCATGCCAGCATCATGACTGCAAAAATGGCATATGTTTTCAGCCAATGGGATCCAACGATTATGTGTGTAAATGTGCACCAGGATATTCTGGAAAACGTTGTGAATATCTTACTAGTCTAAGCTTTGTTCACAACAACTCATTTGTGGAATTGGAACCGCTAAGAACAAAACCGGAGGCAAATGTTACAATTTTATTTGCTACTGAACAAGAAAATGGTGTTTTGCTTTACGATGGCCAAAATGAACATATAGCCGTAGAACTTTTTAATGGTCGCATACGTGTCAGTTACGATGTTGGAAATTATCCCGTCTCAACGATGTACAGCTTCGAAATGGTATCTGACGGAAAGTACCATGTAGCAGAACTTATTGCTATAAAAAAAAA?????????????????????????????????????????????????????????????????????????????????????????????????????????????????????????????????????????????????????????????????????????????????????????????????????????????????????????????????????????????????????????????????????????GAGGAGCAGATGGAGGAAGACAGAGCAGTAATCGAGGATGACGATGTGGAAATGAATGCTTGCCACAATAATCAATGCCGAAGGGGCAGCAAATGCATACCTAAACGGCAAGGAGAGTATGCGTGTCGATGCCGACCTGGATGGGGTGGCCGTTATTGCGAGCAAGCGCCCTCGTGTCGTAAGGAACAGACACGTGAGTATTACATGGAAAATGGTTGTAGATCAAGAAAACCAGTGAAGTTAGCAAAATGTGAAGGAAATTGTGGTTCAAGCTGCTGTCGAGCTCGGAAGACGAAACGTCGTAAGGTGCGACTGATTTGTAACGATGGGACACGTTACACTACGGACGTCGACATTGTGCGTAAATGTTCATGCACCAAAAAGTGCTATTAACGGGCCATAAAAACAATTTCGTAGCCATGCCTGAATATCTAGTACAGGTGGGCCCCAACTAAGCCTAAGCAACCTGTAATTATCAAGCCAGTGTCAGAGTTGCCTACTTTGTAATCACGATTAGCGTCCTTTCACTGTATATATCTTTCTGCTCCTAAACTATTTATTATTACAATTTTACTTTCAGTATGCCTCTACCATGAATCCCCTCATGCACAGCTTTACTGAAGTTTTCATTTTAAGTTCCTCCTGTTCCTCCTACTACCACCACTGCCAAAACTATTTCTACTACCACTGCCCCCCTACACTACAGCCACCTCC

B) Dideoxy sequencing of *G. bimaculatus* *robo 1*

????????????????????????????????????????????????????????????????????????????????????????????????????????????????????????????????????????????????????????????????????????????????????????????????????????????????????????????????????????????????????????????????????????????????????????????????????????????????????????????????????????????????????????????????????????????????????????????????????????????????????????????????????????????????????????????????????????????????????????????????????????????????????????????????????????????????????????????????????????????????????????????????????????????????????????????????????????????????????????????????????????????????????????????????????????????????????????????????????????????????????????????????????????????????????????????????????????????????????????????????????????????????????????????????????????????????????????????????????????????????????????????????????????????TGTTGTGGGCAGTGTATCAGCAAGAGCATCACTGACTGTGCATTCTCCTCCAACATTTATCACAAAACCACAAGATCAGAAAGTAGGTCTGAATGGAATAGCTGTGTTTGAATGTGTAGCAAAAGGCAACCCTCCCCCATCAGTATTTTGGACAAAGGAGGGGAGCCAGGTATTAATGTTCCCTGGGAACTCTTATGGACATCTTCATGTGACTCCTGAAGGTAGTCTTCGAATTCAGGGAGTTCAGCGTGAAGATGCTGGGTTCTTGGTTTGCTCTGCCTTGAGCGTTGCTGGTTCAAATACTGCACGAGCCTTCCTGCAGGTCACATCAGTTGATGATGTGCCTCCTCCAATTGTGCAAATTGGGCCAGTGAACCAGACACTACCATTAAAGTCTGTTGCAACACTTCCATGTCAGGCCACTGGAACTCCTCCTCCAAAAATAAAATGGTACAAAAATGGTTCCCCCCTTGCTGGTCAGGGTCCTAGAATAACAGTTTTAGAAACAGGAACTCTACATATTGATGACTTGCAGTTAACAGATTCTGGCCTGTATACCTGTACTGCTTCATCAGAGAGTGGTGAAACTTCATGGTCTGCTTCATTGACTGTGGAAAAGAATCCTAGTCCTTCAGGCCCAGGTCTTCATCGAG?????????????????????????????????????????????????????????????????????????????????????????????????????????????????????????????????????????????????????????????????????????????????????????????????????????????????????????????????????????????????????????????????????????????????????????????????????????????????????????????????????????????????????????????????????????????????????????????????????????????????????????????????????????????????????????????????????????????????????????????????????????????????????????????????????????AAGTATAATATGGTTACTGTGTTGAATGCTGGAGCTACAAGCTATGCAGTCACCAATTTGCGCAAATACACCAAGTATGAATTCTTCTTGGTTCCATTCTACAAATCAGTTGAAGGTCAACCATCAAACACCAAGAATGTTCAAACTTTGGAAGATGTTCCATCAGCACCGCCAGACAATATTCAAGTTGGAATGATTAACACCACAGCAGCATATGTTAAATGGTCATCTCCTCCACCAGCACATCATAATGGTGTTCTCCTTGGGTACAAGATCCAGGTGAAAGGGAATGGTACAAAAGTGCTTGCCCAGATGACATTAAATGCTTCCACAACTTCTGTGTTGTTGAATAACCTTACAACTGGCGGCGCATATACTGCTCGTGTTGCAGCATACACACGAAGAGGCTTGGGTCCATTCTCTAGTCCAGTTTCTCTCATTATGGACCCTGCATTGCTTCATCAATATCCTCCTAGAGCACATCCAAGTGAAGGAA??????????????????????????????????????????????????????????????????????????????????????????????????????????????????????????????????????????????????????????????????????????????????????????????????????????????????????????????????????????????????????????????????????????AATACAGGTGCTCCAAGCTTGGAGCTAGTTTCGAATACAACTGATTATGCAGAAGTGGATACTCGAAATTTAACTACATTTTACAGTTGTAATCGTAACAATAAAGATAATGTTCCTGAAATACCAGCTCCATATGCAACTACTACACTTATCAATTCCATTCCTCGAAGAGAAATGGACAATGGGCATATGTTTATGCCCATTACAGTGGGAGGTCCTGGGGAAGCTAAGACATCTAGTTCCAGTGATTCGTGCGTGAAACCAGACTTGTCAAGTTTGGACACAAACCCTGAGCCTGGCAATAAATCAAGTAGCCCTAGCTCAGAAGTAGGCAATATGTATGCTGATGATGGAAACATGCAACTTCGTCGGCTACCTTTACATCAACCTCCTTCACAAGTGAGAAAGTTCCCAGTAGGAGGACAGCAAGTACTTCCTAACTGGTCAGAGCTACTTCCTCCTCCGCCAGAGCATCCTCCACCATCTACAATGAATGAAGGCATTTCTAATAATAGGTTGCAGATGCCATCTGGAAGTTCCTCAAATAGGGGAGTATCTCCCAATTTCCATCCTCATACAGGAAATTTCAATCCCAATAGCCCTCTTCTTGGCAAGAGGAACACATGCTCTCGAGAAGGAACACCTCTTGGTCATCTTTCTCATCCTGGTGAGGGAAATCATGCTGTTCCTATGGGCAACACTCCTCCTCTGCCTCCTGTTCGAGGTGGAAGCAGTTGCAGTAGTGCAGGATACACAGGACCTTGGGTCCCCAATAATCCTGCAGAACAGAACATGTATGGCAGCAACTCAGGAGGAAGATATTCCCTTATGCCACCCCAGCAGCACCCTCCACCGGTTCCAAATTTTCCAATGGGCTTTGGAGGAGCTGGAGGCAGTTCAACTGGGGGAAGTAGTAATCACAGTGGATCACAGCACCCTGGCCACCATCACAATCACCACCACCATCATCATCATCACCATGCCAAGAGTCCCAGTGGGAATCCAAATGGAAATCCTCATTTGGAGGAATCAA?TTATGAAAGTGGGTCATTAGTGTATGGAGACACTGGAGGAGGCCATGATGATTATCAGCAGCACAATTCTTCAACAGGAGGTACATCTCAGATTGGCAGTAGTGCAGGTTATGGAGCTATGGACCGAGGAATTCAATCTTCTCTCCCAAGTTTAGCATCGGAAAACCTTTCATCCAGACTGCACCCTAGTGTTGCTGCTCAAATGGCCATGGATCTGGGTAATCCTTCTGATGGCGAAGCAGTAATGGGAGACTATAGTGACTGCGATCGTTGGCGGAGCCCAGGTGGAGAGGATTCCACCACTGCAGGTTCCTGGGATGAAGACCGTGGTAGTTGCAGCAGTGGTGATGCCAGTGATACATGTTGCTCTTGCAGTGAATCAAGCTGCTTATATGCTGAGACAACAGAATTAGCCAATCAGAATCCAGCTGGGCTTCCTCCACAGGTTGGAGGTCCGTGCACACATAATGCAGCTGCAAATGCCCGCCGACATGTTCGGCGACAATACCCACCCCATCGAACATCTGCTTCTGGTTCAGGACGTCCAGTTTCTCCATCCTATAGCACTGATAGTAATTACAGCTGTGCCCGTCCACCACCAGCTCGTAGTCATCTGCGACCAGCTGTTGCTACAAGCACAGCTTCATCTGTAGGTGATTCAAGTCCTTACACAAGTCAGACAGCTACACCACAACATTGTAGAAGAGATGATACTCCAGCTTATGCCAAGCCTAATTATCCAACATCTCAAACATCTCGCAACAATACATTAGGCAGTGGTGGTACTCTAGTGGGTAGTTCAGCTGGCAGTCAACGCCTCAAAAATCTTGGTAATGTGTTCCAGAACTCTTCCTTTCCAAATACAGGTACATCTACTACATCCAGTTCAAGCCC??????????????????????????????????

C) Dideoxy sequencing of *G. bimaculatus* *Sema1* (**Accession No. MF817714**)

CCGATCTCGCCGGCGTGCCGCCCGGCGCCCGAGGCCGCGGCCTCCTCGCCCTGCTGCTGGTGACGATGGGCGCCGCCGCGACCGTGGACGCCGCCACCGGTGCCTGGCAGGAGAACGTGCGTCCCAAGATGTACGTCCAACTAGGCACCAACGATGTGTTTCGCTTCACCGGAAACGACTCTCACACCGACTTCTTCCGGCTGGTGATAAGGGATGGCAACTCTCTTCTCGTCGGAGGAAGGAATCTGGTCCACAACCTGAGTCTGCCAGAGCTTGTTGAAAATCAGCGGCTGGTGTGGTACTCGCCGGACGAGGACGTAAAGATGTGCGTGATGAAGGGCAAGGACGAGGAGAATTGCCAGAACTACATTCGCATCCTGGCACGAACAGGAGCTGGCCGCTATCTCGTCTGCGGCACCAATTCTTTCAAGCCCGTGTGTCGCGACTACTCCATACAGAGTTCTGGTTATGTGGTGGAGAGAGAGAAAAACGGGCAAGCGTTATGCCCCTATGATCCAGCGCAAAATAGCACAGCTGTCTACGTTGACAGCGACCTCTACACGGGGACCGTAGCCGACTTTTCGGGCATGGACCCCATCATCTACAGGGAGCCGCTGCAGACTGAGCAGTACGACTCCATGAGCCTCAACGCTCCCAACTTCGTGAGCTCCATGACGCAGGGAGATTTCGTCTACTTCTTCTTCCGAGAAACTGCAGTGGAGTACATCAATTGTGGAAAGGCGGTGTACTCGCGCGTGGCGCGCGTGTGCAAGTACGACCGCGGGGGCCCGCATCGCTTCCGCAACCGTTGGACCTCCTTCCTCAAGTCGCGCCTCAACTGCTCCGTGCATGGCGACTTCCCCTTCTACTTCAACGAGATCCAGTCGACGACGGAGCTGATCGAGGGCTCGTACGGCGAGACGACGGCGCAGCTGGTGTACGGCGTGTTCACGACGCCGCCCAACAGCATCAGCGGGTCCGCCGTGTGCGCCTTCGCCCTGCAGGACATCACCGACACCTTCGAGGGCAACTTCAAGGAGCAGGCGCAGCTCAACTCCAACTGGCTGCCCGTGCAGAGCGTGAAGCAGGTTCCTGACCCCCGACCGGGCCAGTGTGTCAACGACTCCCGCACTCTGCCTGACCTGACCCTCAACTTTATCAAGACGCACTCGCTCATGGACGAGTCAGTGCCTTCTTTCTTCGGACAGCCCATTGTTATTCGCACCAGTTTTCATTACAGATTCACCCAGATAGCAGTAGATCCCCAAATTAAAACACCTGGAGGCAAGCCTTATGACGTTCTCTTCATAGGAACAGACAATGGCAAAGTCATCAAAGCAGTGAATGCAGATTCTGCTGACAGCAATACTGAAGTGAGTCCCGTTGTTATTGAAGAAATTCAAGTGTTTCCTCCTCATGTAGCAGTACGCAACCTCAAAGTAGTGAGAGATTCTTCATTTGATGATGGACGACTAATTGTCGTGAGCGACAGTGAGGTGCAGTCACTGCGTCTTCATCGCTGTTATAGCGACAAAATTCTTTCTTGCAGTGAATGTGTTGCACTTCAAGACCCATATTGTGCCTGGGACAAACAGTCCCAAAAGTGTCGATCTGTAGGTGCTCCTCGATGGAATGATGAGAAGTACTTTTATCAGAGCATTTCCAAAGGAGTTCATTCTGCTTGCCCTGCCAGTAAAGTAATGGGTAAAGATGCTGGCAGTGTTGGAGGATTGTCATCAAACTATCCCAAGTCATTTAATCATGACTCTGGTCGTTCAAGCAAAGATATACAGGGTGGAGAAGTCATTAACATTATGCATGATGAAGAAGAACACACAGGCCCCGAGGTCAGTGCGGCTGATTCACCAATGCCACAATATTCTGTGGAGACCTTAGCAATGGCAGTTGTAGCAGGGTCAGTGGCAGCATTGGTCGTTGGCTTTGTCACCGGCTATCTATGTGGTCGCAAATGCCACAAAGAAGAAGAAGACAACCTCCCATATCCTGATACTGAGTATGAGTATTTTGAACAAAGGCAGACAGTAAACAGCCGACTGCAACCAGAACCAAAGCTGTTACCACAGGAAGAGGTGACCTACGCAGAGCCAGTGTTGGTCCCCGCCCCAGGGCCCAACAAGCTGAACTCCCCCAAGAGCACCCTCCGGAAAGCACACAACGCCAACCACGCCGCCGAGACCCTGTTTCAGTTCTCCGACAACTACACCCCCCCTCCCCGCGACCCCTACGCGCATCAACGAGGCCGAGACAACTTCGGCACCCTACGCTCCCAGCAAGGCGACGGCTACCGCGGCGGCGGCGGTGGTGGCGGCGGAGGCGGCGGCCGCGCCCCC

D) Dideoxy sequencing of *G. bimaculatus Sema2a* (**Accession No. EF036538**)

GACCATGTACGCGAGTTCTCCTGTGGCAAGATGTACTACCGCACATTCTATTTGGACGAAAGAAGGGATTCCCTCTACGTTGGAGCCATGGATCGAGTTTACCGCCTTAACTTGAGTAACATTAGCCATTCAAATTGCGAGAGAGATTCGATGAGTCTGGAACCGAGCGACGTGGCCAACTGCGTCTCTAAAGGGAAATCGGAGCATTTCGATTGCCGGAATCACATCCGCGTCATTCAGCCAATGGGAGATGGAGGCCGATTGTACATCTGCGGCACCAATGCCCATAGCCCAAAGGATTGGGTTATCTATTCCAACCTGACGCACTTGATGCGCAAGGAGTTCGTGCCGGGCGTGGGGCTGGGCATCGCCAAGTGCCCCTACGACCCGGCTGACAACTCCACGGCGGTGTGGGTGGAGCGCGGCAACCCCGGCGAGCTGCCCGGCCTGTACAGCGGCACCAACGCCGAGTTCACCAAGGCCGACGCCGTCATCTTCCGCACCGACCTGCACAACCTCACCACGGGCCGCTCGGAGTATACCTTCAAGCGCACGCTCAAATACGACTCCAAGTGGCTCGACAAGCCTAACTTCGTGGGGTCGTACGACATCGGGCAGTACGTGCTGTTCTTCTTCCGGGAGACGGCCGTCGAGTACATCAACTGCGGCAAGAGCGTGTACAGCCGAGTGGCGCGCGTCTGCAAGAAGGACACAGGTGGCAAGAACATCCTGTCGCAGAACTGGGCCACCTACCTCAAGGCGCGGCTCAACTGCTCCATCCCGGGCGAGTTCCCCTTCTACTTCAACGAGATCCAGAGCGTGTACAAGGTGCCCGGCGACGACACCCGCTTCTACGGCGTGTTCACGACGTCGATGACCGGGCTGGTGGGCTCGGCCATCTGCGTGTTTACGCTGCGCGACATCCAGGAGGCGTTCCGCGGCAAGTTCAAGGAGCAGGCGACGTCGGCCAGCGCCTGGCTGCCCGTGCTGTCCTCGCGGGTGCCCGAGCCGCGACCCGGCGACTGCGTCAACGACACCGAGACGCTGCCGGACACGGTGCTGAACTTCATTCGATCCCATCCGCTCATGGACGCAGCAGTGGCACACGAGCACGGAAAACCCGTTTTCTTCAAACGCGACCTTCTCTTTACGGATCTCGTAGTGGATCGCCTGAAAATAGACCTCATCGCCTCAATTAAAGAATACATCGTCTACTACGCCGGAACAAATAACGGGCGCGTGTACAAAGTGGTGCAGTGGTACGACGAGGACGCCGAGGACTCGCGCTCGCGCCTGCTCGACA

E) Dideoxy sequencing of *G. bimaculatus* *PlexB*

?????????????????????????????????????????????????????????????????????????????????????????????????????????????????????????????????????????????????????????????????????????????????????????????????????????????????????????????????????????????????????????????????????????????????????????????????????????????????????????????????????????????????????????????????????????????????????????????????????????????????????????????????????????????????????????????????????????????????????????????????????????????????????????????????????????????????????????????????????????????????????????????????????????????????????????????????????????????????????????????????????????????????????????????????????????????????????????????????????????????????????????????????????????????????????????????????????????????????????????????????????????????????????????????????????????????????????????????????????????????????????????????????????????????????????????????????????????????????????????????????????????????????????????????????????????????????????????????????????????????????????????????????????????????????????????????????????????????????????????????????????????????????????????????????????????????????????????????????????????????????????????????????????????????????????????????????????????????????????????????????????????????????????????????????????????????????????????????????????????????????????????????????????????????????????????????????????????????????????????????????????????????????????????????????????????????????????????????????????????????????????????????????????????????????????????????????????????????????????????????????????????????????????????????????????????????????????????????????????????????????????????????????????????????????????????????????????????????????????????????????????????????????????????????????????????????????????????????????????????????????????????????????????????????????????????????????????????????????????????????AGATTTCATAGCCCAACTGGTGACATTCTCCTTCCTAATAATGTGCCAAAGGAGATTGTACTTGAGGTGGATAATCTGCCTCATCCTCAAGTGGGACACACGGGCTTCCAATGTATTGTGAATATTGAAGGAGCCAAAATGATGGTCCCAGCTAGAGTTGAATCGAACCGCTTCATCGTATGTGACAGGACAACTTATTCATATGAAGCCAACACAGGAGAGTATGAAGCAAGTGTTACTATTGTTTGGAATCGTAATCATCATGTGGACACTATAAATGTTACATTATACAAATGTGACATTCTTGGATCACACCGTGAACATCCTGATTGTTCTCTGTGTGTAACACGAAGTGCAAAATACCAGTGTGTTTGGTGTGGATTGACTTGCAGTTATTCTCAAAGTTGCCAGCACATTCCAGTCAGTGAATGCCCTAAACCCAGAATAGATATGATTAAGCCACTCAGTGGGCCTATTGAAGGGGGAACTCTTGTCACAATAGAAGGAAGTAATCTTGGTCTTAAAGAAGAGGATGTGAAAGGAAAAATAAGTATTGGAAGTACTCCTTGTGAACTAGTCCACTATGAAGTATCAGTTCGTATAGTTTGTCGCACAGGTCCTTCAGATGGTGAAATTATTGCTCCTGTCATGGTGGGCAATGAAGCTGGATATACAGAATCTGCTGTTCATTTTAGTTACAAGGATGTTCAACTACTTGGAGTATTTCCTCCAATGGGACCTCAAAGTGGAGGTACCCAATTGGCTATAACTGGCCAATACCTGAATATTGGTAGCAGTATTTCTGCATATCTAGATCATTTGCCGTGTCATGTGAATTCTACACAAGCTTCAAGTAGTCGAATTACGTGCATCACAACTCGTTCAAGTGAACCTATGCGTGTTCAGAGGTTGACCCTTACAATTGATGGAGCTAATCGTACTTTGGAGAGTAATCCTTTCAATTATACTCTTGACCCCACAATAATGGAAATAAAACCCCTCACAAGTTTTGTATCAGGTGGAAGAATGATAACAGTCCATGGAACAAATCTAGACACAATATTAAAGCCTGAAATGGTTGTATACATTGATGAAGAACCCACTCCTATTAACAAAACAGTTTGCAGTGTGCTGAATACTGCACAAATGGAATGTCCCTCTCCGCCAGTGAATCGAAAGTTTCTTCTTAGTGCAAGACAACGTCGCTCTCCAAATAAACCTTCCCGCTCCATGCCAAAATATGGAACTGATGCTCAATTGCAATTGCGAATAGGTTTTATTATGGATAATGTTGAATCAGTTAAAGATTTGGAGAAACATTTCCAAAATCTTCGTTCACATTTGCTGTATGTTGACGACCCACAATTCTTCCGTTTTCCAAACATGAACAAACTTTACAAAGGAGACACATTGGTTATAGAGGGAGTGAATTTGAACCTTGCCAGTGATGAATCAGACGTAAATGTTACAATTGGCACCCAACCTTGTAATGTTACATCCTTGGCATCAACACAGCTGGTGTGCAGCCCACCTGAAGTTCAGCCAGCAGGCACTGATGAAATTGGTGTTAGGACTGATACTAATCTCCCATTAGT??????????????????????????????????????????????????????????????????????????????????????????????????????????????????????????????????????????????????????????????????????????????????????????????????????????????????????????????????????????????????????AGCATTTGCTGAACTCCAGACAGATATGACTGACCTCACTGCAGATTTAGAATCTTCAGGCATTCCTACCTTGGATCATAAAAATTACATTATGAAAGTCTTCTTCCCAGGTGTTAGTGATCATCCTATTTTAAATGATCCCAAGATTAGAGTGAATGGTCCAAGAACAAACTATGATGCAGCAATGTTGCAATTTGAACAGCTGATTAATAACAAATACTTTATATTGACTTTTATTGAGACTTTAGAAGCACAAAAATCTTTCAATATACGGGACAAAGTGAATGTTGCATCATTACTAATGGTGGTACTAATGGGCAAAATGGAATATGCCACTGATGTGTTGTGTAGCTTACTTCTGCGCCTAATTGATAAATCTGTGTGCACCAAACATCCTCAGCTAATGTTGAGAAGAACGGAATCAGTTGTCGAAAAGATGTTGACAAACTGGATGGCACTTTGCATGTATAATTATTTAAAAGATTATGCTGGTTCATCATTAT????????????????????????????????????????????????????????????????????????????????????????????????????????????????????????????????????????????????????????????????????????????????????????????????????????????????????????????????????????????????????????????????????????????????????????????????????????????????????????????????????????????????????????????????????????????????????????????????????????????????????????????????????????????????????????????????????????????????????????????????????????????????????????????????????????????????????????????????????????????????????????????????????????????????????????????????????????????????????????????????????????????????????????????????????????????????????????????????????????????????????????????????????????????????????????????????????????????????????????????????????????????????????????????????????????????TTTTATCAAGAACCCAGACTTTATCTTTGACATTAATAAAACAACAACAGTAGACTCATGCTTGTCCGTGATTGCTCAAACATTCATGGATTCTTGCTCTACAACTGAACACCGTCTTGGAAAAGATTCTCCATCTAATAAGTTACTGTTTGCCAAGGATATTCCCCATTATCGAGAAATGGTGTCTCATTTTTACTGTGACGTCCAAATTCTACCCCAAATCACAGATCAAGAAATGAGTACAGCCATGCAGCAGTTATCTATTTCACAATTGGGTGAATTTGACACTGTATCAGCGCTCAAGGAACTTTACATCTATGTTACAAAGTACAGTGACCAGATCCTGGATGCACTGGATGTTGACCCCTATTGCAAGAAAATGCATTTAGCACACAAATTGGAAAATGTGGCCTGCACTCTTGAAGGAGAGGAGACATCAACATGCTGA
